# Chaperone directed heterobifunctional molecules circumvent KRAS^G12C^ inhibitor resistance

**DOI:** 10.1016/j.canlet.2025.217691

**Published:** 2025-04-07

**Authors:** Ines Pulido, Qiyue Luan, Sara Pastor-Puente, Laura Gunder, Yaya Wang, Chenghao Ying, Jinhua Li, Yuetong Sun, Yan Dai, Christian Ascoli, Khaled Abdelhady, Malek Massad, Thomas L. Prince, Guoqiang Wang, Kevin P. Foley, Weiwen Ying, Ian Papautsky, Julian Carretero, Takeshi Shimamura

**Affiliations:** a Department of Surgery, Division of Cardiothoracic Surgery, University of Illinois Chicago, Chicago, IL, 60612, USA; b University of Illinois Hospital & Health Sciences System Cancer Center, University of Illinois Chicago, Chicago, IL, 60612, USA; c Department of Biomedical Engineering, University of Illinois Chicago, Chicago, IL, 60612, USA; d Department of Ophthalmology and Visual Science, University of Illinois Chicago, Chicago, IL, 60612, USA; e Ranok Therapeutics, Waltham, MA, 02451, USA; f Ranok Therapeutics, Hangzhou, 310020, China; g Department of Medicine, Division of Pulmonary, Critical Care, Sleep and Allergy, University of Illinois Chicago, Chicago, IL, 60612, USA; h Department of Physiology, Universitat de Valencia, Valencia, 46100, Spain

**Keywords:** KRAS, Resistance, G12C, HSP90, NSCLC

## Abstract

While KRAS^G12C^ inhibitors have shown promising results in clinical activity, acquired resistance remains a significant barrier to durable responses. Combination therapies have been explored to improve the efficacy of KRAS^G12C^ inhibitors; however, their use is often restricted due to toxicity and limitations in clinically amenable dosing schedules. Transcriptomic profiling and functional assays on acquired resistant models to adagrasib identified an enrichment of HSP90 client proteins in resistant phenotypes, suggesting a therapeutic vulnerability. To address the finding, RNK07421, a novel heterobifunctional molecule, was developed to simultaneously target KRAS^G12C^ and HSP90-client oncoproteins. Structural and biochemical analyses demonstrated that RNK07421 disrupts KRAS^G12C^ interactions by inducing a non-natural interface with HSP90, thereby impairing oncogenic signaling. In vitro, RNK07421 effectively suppressed ERK reactivation and reduced viability in KRAS^G12C^-mutant cell lines exhibiting either intrinsic or acquired resistance. *In vivo*, RNK07421 significantly reduced tumor burden in xenograft models, outperforming both monotherapies and combination therapies. These findings highlight dual KRAS^G12C^ and HSP90 inhibition as a promising strategy to overcome resistance in KRAS^G12C^-driven cancers.

## 1. Introduction

*KRAS* (Kirsten rat sarcoma viral Homologue) is the most frequently mutated oncogene in human cancer. Glycine to cysteine mutations in codon 12 of KRAS, known as KRAS^G12C^ mutations, are highly prevalent, accounting for 14 %, 4 %, and 2 % of NSCLC, colorectal cancer, and pancreatic cancer cases, respectively [1]. Even with a considerable number of KRAS mutations observed in clinical settings, KRAS was deemed “undruggable” until the recent FDA approval of two distinct, highly selective, covalent inhibitors, sotorasib and adagrasib, for treating NSCLC patients with oncogenic KRAS^G12C^ mutation. These inhibitors have shown significant therapeutic efficacy and have shifted the paradigm for the treatment of KRAS mutant tumors [2,3].

Despite the initial effectiveness of sotorasib and adagrasib in the tumor response, acquired drug resistance rapidly develops, highlighting the need for alternative therapeutic strategies to prevent or overcome resistance [4]. Research indicates that KRAS^G12C^ tumors employ multifaceted mechanisms to resist drug treatment, such as attenuating drug inhibition via KRAS amplification, acquiring new mutations within the KRAS oncogene, or activating alternative yet unidentified signaling pathways [5–7]. It is theorized that intracellular heterogeneity or intercellular variability within tumors represents another factor contributing to resistance to KRAS^G12C^ inhibitors [8]. Additionally, non-genetic mechanisms may play a role in drug resistance, including histological transformations in NSCLC [3] or reconfiguration of the proteostasis network by drug-tolerant cells to evade drug inhibition [9]. Due to the considerable complexity surrounding resistance, there is a significant need to study alternative strategies to circumvent resistance and develop novel therapeutic treatments for patients with drug-resistant tumors.

Effective combination therapies that target the activation of alternative pathways to induce proliferative signaling require thorough investigation. The mechanisms driving alternative pathway activation vary considerably among patients with NSCLC and within tumors originating from the same patient [10]. Ongoing clinical trials aim to improve the efficacy of sotorasib and adagrasib by implementing combination therapies with EGFR, MET, or SHP2 inhibitors in cancers [11, 12]. However, this approach may not be efficacious for highly heterogeneous tumors. Furthermore, combination treatments often face dose-limiting toxicities and can lead to the development of new resistance pathways [13,14]. A potential way to ameliorate these problems is through the use of an inhibitor that simultaneously targets multiple oncogenes.

Chaperone proteins play a crucial role in ensuring the conformational maturation and stability of numerous proteins essential for cancer cell survival. Thus, researchers have explored the use of heat-shock protein (HSP) inhibitors as broad-spectrum inhibitors to target oncogenic kinases [15–17]. The HSP90 chaperone inhibitor pimitespib has demonstrated effectiveness in the treatment of gastrointestinal stromal tumors (GIST) and has been approved for use in cases of GIST progression after chemotherapy in Japan [18]. We postulated that combining HSP90 inhibitors with KRAS^G12C^ inhibitors could result in a synergistic effect and allow the suppression of a variety of kinases that drive alternative pathways responsible for conferring resistance.

Here, we demonstrate the utility of a novel heterobifunctional chaperone-mediated protein inhibitor that concomitantly targets HSP90 and KRAS^G12C^ to circumvent *de novo* and acquired resistance to KRAS^G12C^ inhibitors in NSCLC and pancreatic cell lines and NSCLC patient-derived organoid (PDO) models.

## 2. Materials and methods

### 2.1. NSCLC cell lines and STR assays

NCI-H358, NCI-H2030, NCI-H1792, and NCI-H23 (henceforth H358, H2030, H1792 and H23, respectively) and MIA PaCa-2 cells were obtained from ATCC and maintained according to ATCC specifications. To generate the mass-culture of sotorasib or adagrasib-resistant cells, sensitive cells were gradually exposed to increasing concentrations of the respective inhibitors over a period of 6 months in a manner similar to a previously described method [19]. All resistant cells proliferated normally in the presence of 10 μM sotorasib or adagrasib. Methods to generate KRAS^G12C^-resistant cell lines, as well as results from STR profiling, are provided in Supplementary Materials and Methods.

### 2.2. Patient-derived organoids (PDOs)

Deidentified F231 and F631 KRAS^G12C^ PDOs were obtained from the NCI Patient-Derived Models Repository (PDMR) and maintained as described in Supplementary Materials and Methods. RLUN029 KRAS^G12C^ PDOs derived from a deidentified NSCLC patient were developed at Fukushima Medical University, obtained from Summit Pharmaceuticals Inc. (Tokyo, Japan), and maintained as recommended in low-attachment flasks [20].

### 2.3. Agarose microfluidic microwell fabrication

Organoid viability was evaluated in agarose microwell microfluidic devices 200 μm in diameter and 75 μm deep by casting on polydimethylsiloxane (PDMS) replicas of 3D printed masters. This method created 1000x U-shaped microwells that allowed the normal growth of PDO [21]. The PDMS replicas were stamped on 2 % agarose, after which the stamped was removed, forming wells at the bottom of each plate well. The microarrays were sterilized with 70 % isopropyl alcohol (IPA) and rinsed with PBS before any experiments.

### 2.4. Cell viability assays and cell counting

Live cells were counted using the Countess 3 Automated Cell Counter (Thermo Fisher Scientific), and an equal number of live cells were seeded in each assay to compare cell growth kinetics using the Cell Counting Kit-8 (CCK-8) colorimetric assay (Dojindo), as previously described [22]. The results were analyzed and graphed using GraphPad Prism Version 10.1.1 (270).

### 2.5. Western blot analysis

Lysate preparation and western blotting were performed as described previously [22]. A list of antibodies can be found in the Supplementary Materials and Methods section.

### 2.6. RAS activity assay

Treated or untreated cell lysates were prepared according to the manufacturer’s protocol using an Active RAS Detection Kit (#8821, Cell Signaling Technology). Details of the RAS pulldown assays are available in Supplementary Materials and Methods. Lysate protein concentrations were measured using the Pierce BCA Protein Assay Kit (#23225, Thermo Fisher Scientific) prior to active RAS pulldown. Equal volumes of elutes from each active RAS detection kit column were resolved by western blotting.

### 2.7. Proteome Profiler Human Phospho-RTK array kit

Proteome Profiler Human Phospho-RTK Array Kit (#ARY001B, R&D Systems) was used according to the manufacturer’s guidelines. Cell lines and PDOs were treated with vehicle or drug for 72 h before harvesting the samples. Whole-cell lysate (500 μg) was used for each array. The assays were performed according to the manufacturer’s instructions, and the blots were scanned using the iBright 1500 Imaging System (Thermo Fisher Scientific) for densitometric analysis using QuickSpots Software (Ideal Eyes Systems, Inc.). The obtained values were log2 transformed and heatmaps were generated using Morpheus (https://software.broadinstitute.org/morpheus).

### 2.8. Hsp90α FP assay

The FP assay is based on competitive binding of the Bodipy-tagged HSP90 inhibitor to the Hsp90α protein. The HSP90 inhibitor Bodipy was synthesized by Ranok Therapeutics. Briefly, 50X compound working solutions with serial 2.5-fold dilutions were evaluated in duplicates, then Geldanamycin-Bodipy (mix with 4 mM DTT, 0.2 mg/ml BSA) and of Hsp90α working solution were added per well. The plates were then incubated at 25 °C for 300 min. Luminespib was used as the positive control in this assay. Data were read using a PerkinElmer Envision plate reader.

#### 2.8.1. Homogeneous time-resolved fluorescence (HTRF) assay. KRAS_G12C_/SOS1 binding assay

The HTRF assay (Cisbio) was performed in accordance with the manufacturer’s instructions. Compound working solutions with serial 3-fold dilutions were evaluated in duplicate. The reaction was initiated by adding working solutions to a 384-well plate, followed by Tag1-SOS1 and Tag2-KRASG12C. Samples were incubated at 25 °C for 15 min, and then the conjugate pre-mixture (with anti-Tag1-Tb^3+^ and anti-Tag2-XL665) was incubated at 25 °C for 3 h. The data were read on a PerkinElmer Envision with a laser as the light source.

### 2.9. RNA/DNA extraction and cDNA synthesis

RNA from cell lines and organoids was extracted and purified using QIAshredder cell-lysate homogenizers and an RNeasy Plus Mini Kit (#79656, #74134, Qiagen) according to the manufacturer’s instructions. Total RNA was transcribed into cDNA using a High-Capacity RNA-to-cDNA Kit (#4387406, Thermo Fisher Scientific). DNA was extracted using a DNeasy Blood & Tissue Kit (#69504, Qiagen). The samples were quantified using a NanoDrop ND-2000 Spectrophotometer followed by Qubit (Thermo Fisher Scientific).

### 2.10. Expression profiling of adagrasib resistance with RNA sequencing

Adagrasib-sensitive and-resistant NCI-H358 cells were treated with vehicle (DMSO) or 100 nM adagrasib for 24 or 72 h. Total RNA was extracted and quantified as previously described. The samples were sequenced using the Illumina NovaSeq 6000 System at the NUSeq Core (Center for Genetic Medicine, Northwestern University). Methods to utilize RNA-seq data for gene expression are detailed in Supplementary Materials and Methods.

### 2.11. Murine drug treatment studies

All animal treatment studies were reviewed and approved by the Institutional Animal Care and Use Committee of the University of Illinois, Chicago. H2030 or RLUN029 tumor cells (5×10^6^) were grafted onto Nu/Nu or NOD scid gamma (NSG) mice, respectively. Tumor size was measured using the formula V_T_ = 0.5xLxW^2^, and the mice with grafted tumors were randomized into one of the following treatment groups. Mice were treated three days per week with either vehicle (oral gavage), adagrasib (50 mg/kg, oral gavage), capmatinib (10 mg/kg, oral gavage), the combination of both or RNK07421 (100 mg/kg, i/v) for 21 days.

### 2.12. Histology and immunohistochemistry

Harvested tumors were fixed in 4 % formalin overnight at room temperature. The specimens were then embedded in paraffin, sectioned (5-μm thick), and placed on slides for staining. Detailed procedures and a list of antibodies used for staining are available in the Supplementary Materials and Methods.

### 2.13. Statistical analysis

Unless otherwise stated, comparisons of statistical significance were performed using ANOVA and Tukey’s multiple comparison test or *t*-test, where applicable. Statistical significance was set at P < 0.05.

## 3. Results

### 3.1. Acquired resistance to KRAS^G12C^ inhibitors in NSCLC

**Fig. 1.**
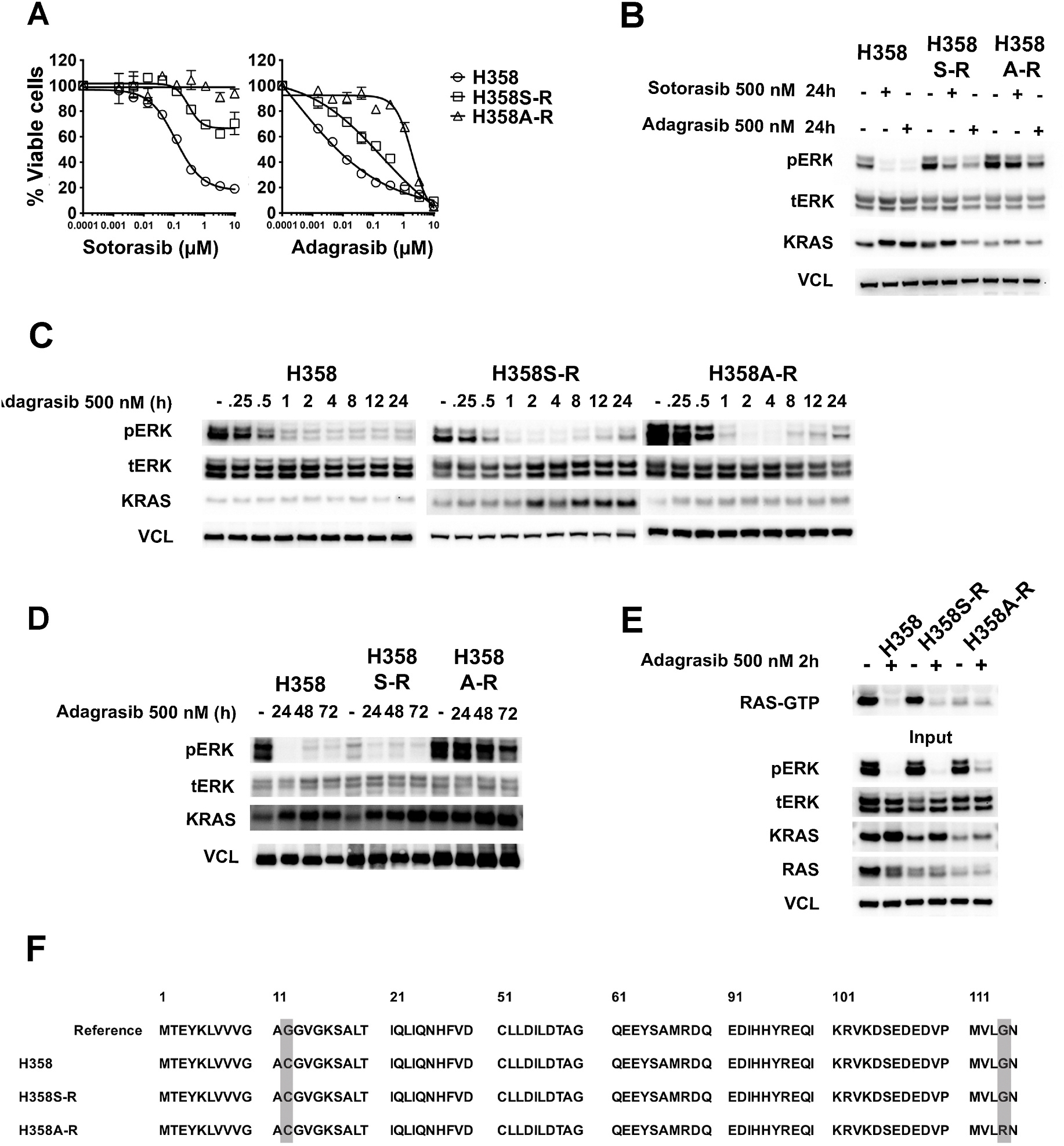
Sotorasib and adagrasib NSCLC resistant cells behave differently to KRAS G12Ci. (A) Colorimetric *in vitro* proliferation assay performed after 72 h of treatment with varying concentrations of sotorasib (left) or adagrasib (right) in the three H358 cell models: H358 parental, H358 S-R (sotorasib resistant) and H358 A-R (adagrasib resistant). Error bars: S.D. Representative experiment of 2 different experiments. (B) Immunoblot showing H358, H358S-R, H358A-R treated with sotorasib or adagrasib for 24 h. Representative immunoblot from three independent experiments. Vinculin (VCL) was used as a loading control. (C) Immunoblot highlighting H358, H358S-R, H358A-R after varying durations of treatment with adagrasib. Representative immunoblots from three independent experiments are shown. Vinculin (VCL) was used as a loading control. (D) Immunoblot highlighting H358, H358S-R, H358A-R after treatment with adagrasib for either 24, 48 or 72 h. Representative immunoblots from three independent experiments are shown. Vinculin (VCL) was used as a loading control. (E) Sensitive and G12Ci resistant H358 cells were treated for 2 h with adagrasib and both specific KRAS, total RAS activity and total input were evaluated. Representative immunoblots from 2 independent experiments are shown. (F) KRAS gene sequence from the three H358 cell lines. The codon 12 substitution (driving G12C mutation) and codon 114 are highlighted.

NCI-H358 (H358), an NSCLC cell line sensitive to KRAS^G12C^ inhibitors, was cultured for six months using increasing concentrations of inhibitors until cells developed acquired resistance to sotorasib or adagrasib (H358S-R and H358A-R, respectively), as confirmed by MTS assays (Fig. 1A). Similarly, MIA PaCa-2 pancreatic carcinoma cells were grown resistant to sotorasib (MIA PaCa-2S-R) or adagrasib (MIA PaCa-2A-R) (Fig. S1A).

After verifying resistance, all cell lines were treated with sotorasib or adagrasib (500 nM) for 24 h to evaluate ERK phosphorylation, a surrogate marker of KRAS-MAPK pathway activation. Sensitive parental cell lines exhibited a marked decline in pERK with either sotorasib or adagrasib treatment, whereas pERK was minimally reduced in resistant cells compared to untreated cells (Fig. 1B and S1B). To assess the timeframe in which ERK reactivation occurred after treatment, cells were treated with adagrasib at multiple time points between 0 and 24 h. Adagrasib suppressed ERK phosphorylation in H358 cells after 24 h, whereas minimal activation was observed in H358S-R cells and H358A-R cells between 12 h and 24 h of treatment (Fig. 1C). This ERK reactivation suggests that drug resistant cells may restore proliferative signals early after drug inhibition.

Adagrasib continued to suppress pERK in H358 sensitive and sotorasib cells for 72 h, but not in adagrasib-resistant cells (Fig. 1D). In all three MIA PaCa-2 cell lines (MIA PaCa-2, MIA PaCa-2S-R, and MIA PaCa-2A-R), pERK initially decreased following adagrasib treatment, but began to rebound after 24-h mark of treatment (Fig. S1C). ERK reactivation was the most prevalent in MIA PaCa-2 G12Ci resistant cells (Fig. S1D).

We then sought to investigate whether adagrasib would directly inhibit RAS activity in drug-resistant cells. RAS activity assays, which measure active GTP-bound RAS in cells, showed a reduction in RAS activity in resistant cells as compared to their parental counterparts. This was observed in both NSCLC and pancreatic KRAS^G12C^ inhibitor cell lines, suggesting a reduced dependence on KRAS signaling in drug-resistant cells (Fig. 1E and S1E). Moreover, KRAS activity was further diminished by adagrasib treatment for 2 h in the input samples.

Given that frequent emergence of secondary KRAS mutations confer adagrasib resistance in NSCLC patient samples [23], we sequenced KRAS to check for the presence of secondary mutations [5,24]. None of the drug-resistant cell lines showed any previously described secondary mutations, and only the G114R mutation was detected in two of the resistant models (Fig. 1F and S1F), which prompted us to further evaluate the non-genetic mechanisms of acquired drug resistance in our cell models.

### 3.2. Resistance to KRAS^G12C^ inhibitor is driven by vertical activation of RTKs

**Fig. 2.**
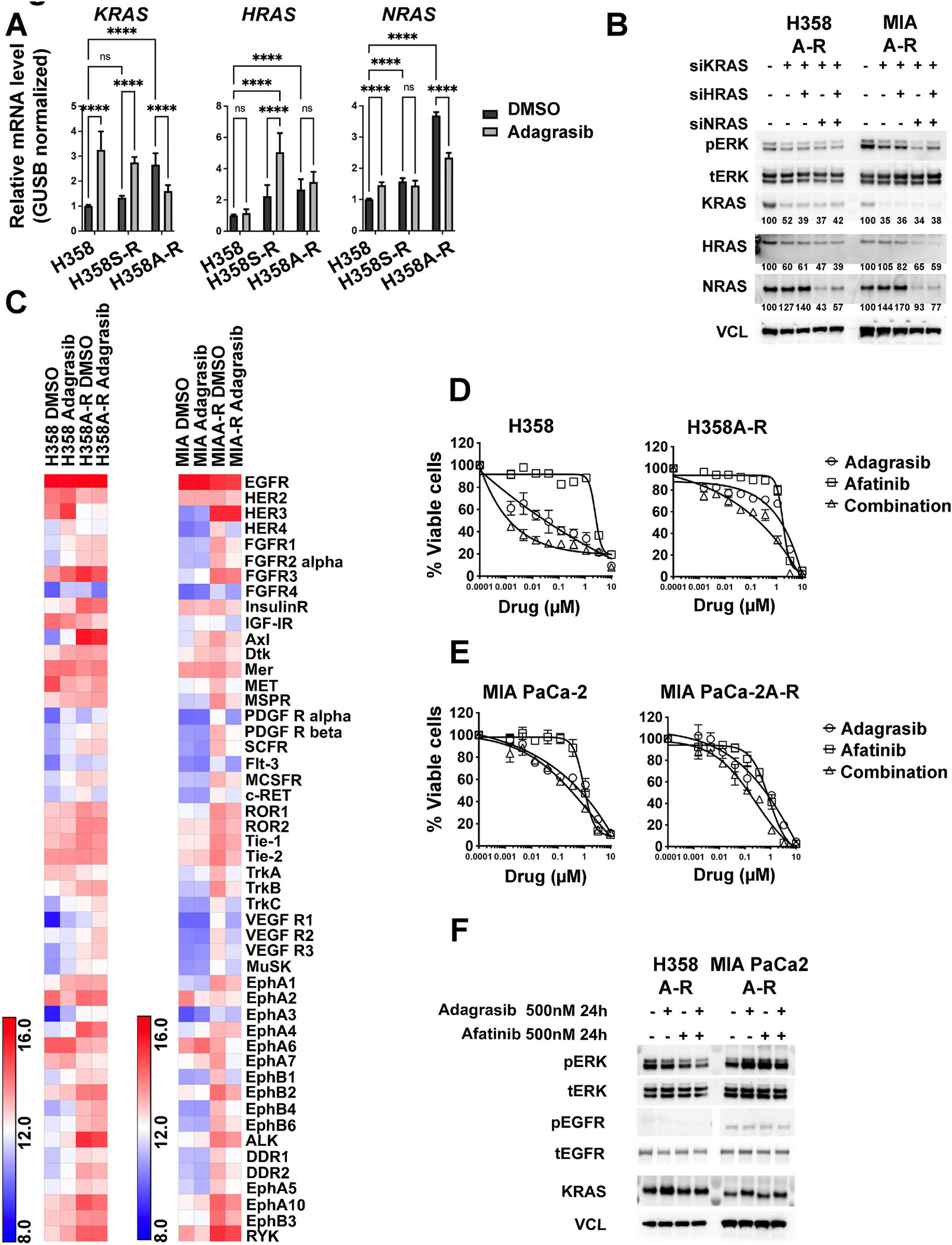
Acquired resistance cells can overcome KRAS inhibition via the activation of numerous RTKs. (A) Quantification of KRAS, HRAS or NRAS mRNA levels in H358 sensitive, sotorasib and adagrasib resistant cells treated with adagrasib or control (DMSO). Error bars: S.D; One-way ANOVA. N.S. Not significant, **P < 0.01, ***P < 0.001, ****P < 0.0001. Representative results from two independent experiments (B) Immunoblot of adagrasib resistant cells treated with or without siRNA for KRAS, HRAS, and NRAS for 72 h. Repression of each siRNA to siControl is shown as percentage. (C) Heatmap highlighting the RTKs as measured by Proteome Profiler Human Phospho-RTK Array in H358, and MIA PaCa-2 sensitive and adagrasib-resistant cells treated with vehicle (DMSO) or adagrasib. (D) Colorimetric *in vitro* proliferation assay performed on cells after 72 h treatment with adagrasib and afatinib in sensitive (left) or adagrasib resistant H358 cells (right). Error bars: S.D. Representative image from two independent experiments. (E) Colorimetric *in vitro* proliferation assay performed on cells after 72 h treatment with adagrasib and afatinib in sensitive (left) or adagrasib resistant MIA PaCa-2 cells (right). Error bars: S.D. Representative image from two independent experiments. (F) Immunoblot showing H358A-R and MIA PaCa-2A-R after treatment for 24 h with adagrasib, afatinib, or in combination. Representative immunoblots from three independent experiments are shown. Vinculin (VCL) as a loading control.

Overexpression of wild-type RAS isoforms has been reported as a mechanism of adaptive resistance to KRAS^G12C^ inhibitors, driven by RTK-mediated activation of the wild-type RAS [7,25]. To evaluate whether the most common isoforms of RAS compensate for KRAS inhibition, mRNA from the 72-h treated H358 cell lines was assessed by RT-qPCR. Interestingly, adagrasib treatment increased KRAS and HRAS mRNA levels in H358 and H358S-R cells but not in H358A-R cells. In H358 cells, adagrasib treatment led to a significant increase in NRAS RNA levels, as opposed to H358A-R cells, in which NRAS mRNA significantly declined after treatment (Fig. 2A). In each of the three pancreatic cell lines, adagrasib treatment led to an increase in KRAS mRNA levels, with a particularly notable elevation observed in MIA PaCa-2S-R and MIA PaCa-2A-R cell lines. However, *HRAS* and *NRAS* mRNA levels only increased in MIA PaCa-2S-R cells after adagrasib treatment (Fig. S2A).

As most RAS isoforms tend to be augmented after adagrasib treatment, we aimed to assess the impact of genetically repressing several RAS isoforms in adagrasib-resistant cells. Depletion of KRAS alone resulted in a limited reduction in the phosphorylation of ERK, and the concurrent depletion of KRAS with HRAS or NRAS did not further decrease ERK phosphorylation in H358A-R cells (Fig. 2B). In MIA PaCa-2A-R cells, the concurrent depletion of KRAS and NRAS resulted in the most dramatic reduction in pERK levels compared to untreated and other depleted cells (Fig. 2B).

We previously confirmed that adagrasib treatment increases RAS transcription in these cell models; however, receptor tyrosine kinases (RTKs) can independently activate transcription in the absence of RAS [7,26]. To assess how cells may bypass KRAS inhibition, we profiled the activated RTKs in KRAS^G12C^ inhibitor-resistant cells. Exposure of NSCLC H358 cells to adagrasib treatment resulted in the activation of ErbB2, 3, and 4, whereas MIA PaCa-2 cells activated EGFR (ErbB1) and ErbB2 (Fig. 2C and S2B). Interestingly, the adagrasib-resistant cells before and after adagrasib treatment promoted the global activation of almost all RTKs evaluated, not specific families (Fig. 2C and S2B).

Given these results, we sought to evaluate combination treatments, specifically drugs that could potentiate a synergistic effect when combined with adagrasib. As EGFR family members were consistently phosphorylated in all models, we hypothesized that adagrasib treatment in combination with afatinib, a known EGFR family inhibitor, would increase drug sensitivity. This combination treatment resulted in a minor reduction in cell viability (Fig. 2D and E) and effectively suppressed ERK phosphorylation in parental-sensitive H358 and MIA PaCa-2 cells compared to adagrasib treatment alone (Fig. S2C), adagrasib-resistant (A-R) cells showed greater diversity in RTK activation (Fig. S2B), and dual EGFR-KRAS inhibition treatment did not significantly sensitize cells to adagrasib (Fig. 2D and E), as evidenced by sustained pERK levels after treatment (Fig. 2F). Subsequently, we tested the combination of adagrasib and AEE788, a multi-RTK inhibitor of EGFR, ErbB-2, and VEGFR-2, in adagrasib-resistant cells. This combination only reduced pERK levels in H358A-R cells compared with the control and individual treatments (Fig. S2D) and showed no reduction in viability between the combination treatment compared to adagrasib alone (Fig. S2E). These results suggest that the elimination of cells with acquired resistance might require the concomitant inhibition of several RTK targets.

### 3.3. Widespread potential for the RTK pathway-driven innate resistance to KRAS^G12C^ inhibitors

**Fig. 3.**
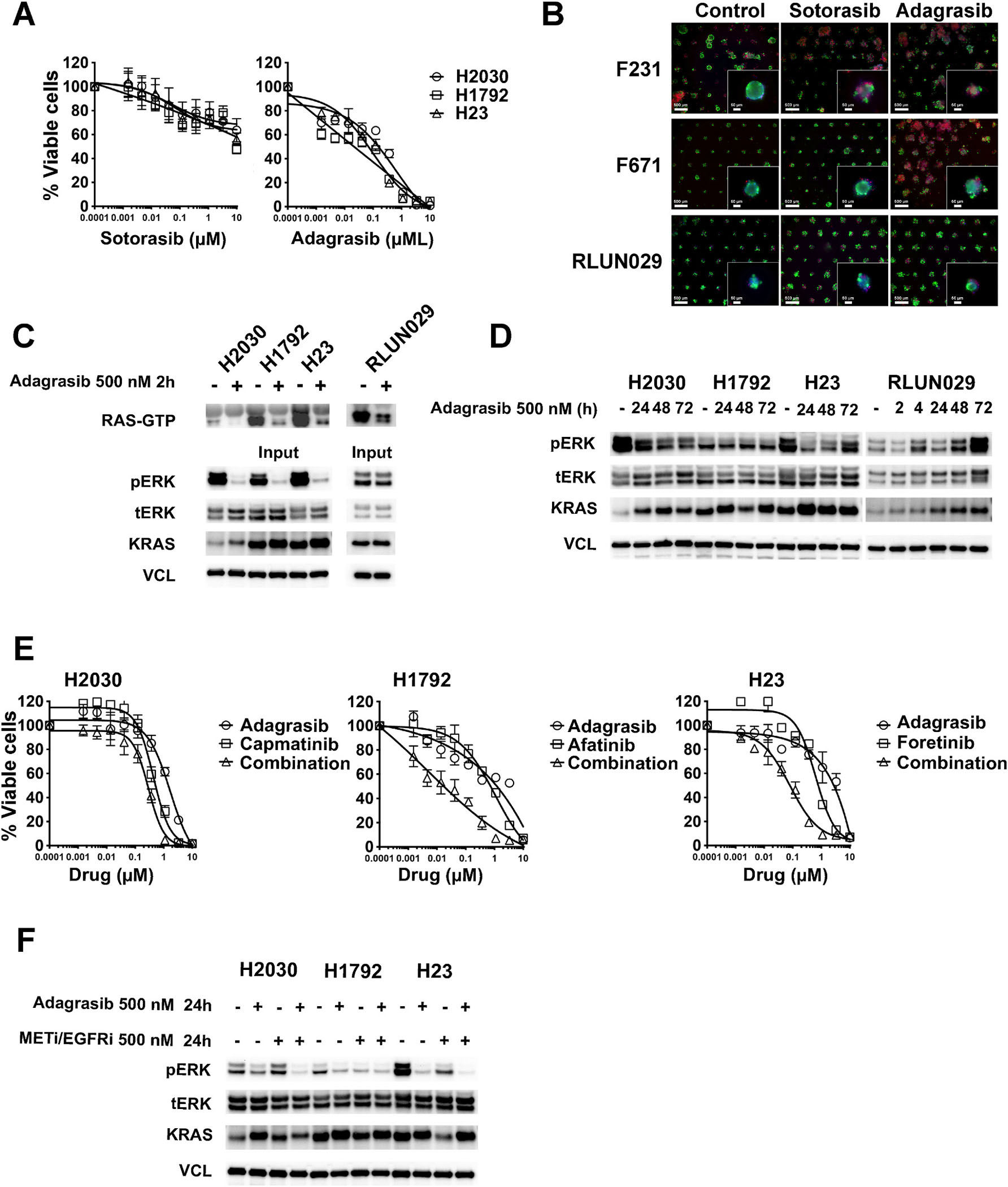
Innate resistance to G12Ci in cell lines and PDOs. (A) Colorimetric *in vitro* proliferation assay performed after 72 h of sotorasib (left) or adagrasib (right) treatment in H2030, H1792 and H23 cell lines. Error bars: S.D. Representative image from two independent experiments. (B) Live/dead staining images of F231, F261 and RLUN029 NSCLC KRAS^G12C^ PDOs after 7 h of treatment of the indicated inhibitor. Live cells in green (calcein AM), nuclei in blue (Hoechst 33342), and dead cells in red (propidium iodide). Each images includes a representative image of a single spheroid in each microarray device. Scale bar 500 μm, inlet 50 μm. (C) H2030, H1792, H23 cell lines and RLUN029 PDOs were treated for 2h with adagrasib, and specific KRAS, total RAS activity, and whole lysates were evaluated. (D) H2030, H1792, and H23 cell lines and RLUN029 PDOs were treated with 500 nM adagrasib or left untreated for the annotated hours. The immunoblots highlight that pERK reactivation occurs in a time-dependent manner. Representative immunoblots from two independent experiments are shown. Vinculin (VCL) was used as a loading control. (E) Colorimetric *in vitro* proliferation assay after 72 h of adagrasib combination treatments: H2030 with capmatinib, H1792 with afatinib, and H23 with foretinib. Error bars: S.D. Representative image from two independent experiments (F) Immunoblot showing that the above-mentioned drug combinations with adagrasib (H2030, capmatinib; H1792, afatinib; H23, foretinib) only slightly reduce pERK levels. Representative immunoblots from three independent experiments are shown. Vinculin (VCL) as a loading control.

After evaluating the cell lines in which we induced drug resistance, we aimed to assess cells that would recapitulate innate resistance, consistent with cases observed in clinical settings. Innate resistance to sotorasib and adagrasib was observed and confirmed in several cell lines (H2030, H1792, H23; Fig. 3A) and patient-derived organoids PDOs (Fig. 3B) harboring the KRAS^G12C^ mutation. We analyzed three different PDOs harboring the KRAS^G12C^ mutation (Figs. S3A and S3B) with variable drug sensitivity, from fairly sensitive (F231 and F671) to resistant (RLUN029) (Fig. 3B and S3C). Remarkably, treatment with adagrasib rapidly reduced RAS activity within hours in these innate resistance models (Fig. 3C).

Although adagrasib treatment can reduce RAS-GTP, even in innate resistant models, we observed that adagrasib treatment did not reduce ERK phosphorylation for extended treatments periods, as evidenced by activated pERK signaling 24-h post-treatment (Fig. 3D). This pERK reactivation after drug inhibition was sustained by increased levels of all RAS isoforms but predominantly by KRAS in both innate resistant cells and PDOs (Fig. S3D). The analysis of alternative RTK-mediated proliferation signals indicated that RTK activation was lineage-dependent, and that each of the innate resistant models and PDOs activated certain pathways: ErbB2 (H2030, H1792, F231 and RLUN029), MET (H23), and ROR1 (F671) (Fig. S3E). Accordingly, drug combination treatments were designed to suppress KRAS^G12C^ and the main RTK involved in surpassing drug inhibition in the innate KRAS^G12C^ resistant models which resulted in increased cell death (Fig. 3E) and sustained reduction in pERK levels (Fig. 3F).

### 3.4. RNA-seq profiling of KRAS^G12C^ inhibitor resistant cells reveals a vulnerability to HSP90 inhibitors

**Fig. 4.**
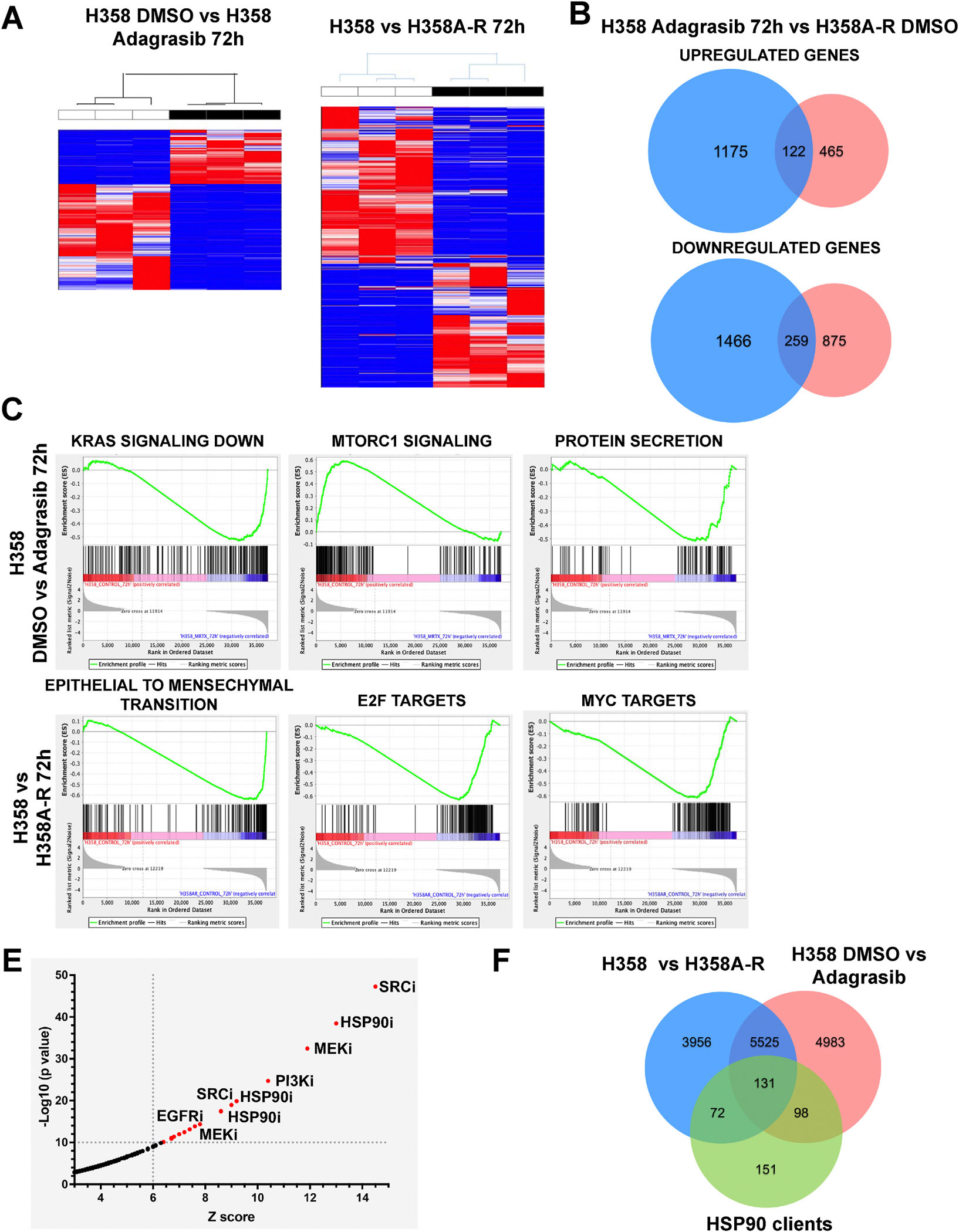
Adagrasib resistance remodels the transcriptomic profile. (A) Heat maps of the differential gene expression profiles for adagrasib sensitive and resistant cells as assessed by transcriptomic sequencing. (B) Venn diagram illustrating the overlapping upregulated and downregulated genes in parental cells treated with adagrasib and H358A-R cell treated with vehicle (DMSO). (C) Gene Set Enrichment plots detailing enriched transcriptomic signatures in H358 cells adagrasib-treated for 72 h compared to vehicle-treated or in H358A-R cells vs sensitive H358 cells. (D) Gene Set Enrichment plots detailing enriched transcriptomic signatures in H358 adagrasib resistant cells vs H358 adagrasib sensitive cells. (E) iLINCS analysis of drug vulnerabilities associated with the adagrasib resistant signature. (F) Venn diagram illustrating the overlapping upregulated genes in parental cells after adagrasib treatment, H358A-R cells compared to parental cells and HSP90 clients.

Transcriptomic differences between drug-sensitive and adagrasib-resistant cells are numerous and complex. To fully understand the alterations underpinning drug resistance in H358 cells, we evaluated the differentially expressed genes in adagrasib-sensitive and adagrasib-resistant cells treated with adagrasib via RNA-seq. Unsupervised clustering of significantly expressed genes in H358 cells exposed to DMSO or adagrasib for 72 h suggested that the transcriptomic alterations after adagrasib treatment were substantial, upregulating the expression of hundreds of genes (logFC>5) (Fig. 4A, left). These differences were even more pronounced when comparing the genes that facilitated the transition from the adagrasib-sensitive to adagrasib-resistant phenotype (Fig. 4A, right). Acute adagrasib treatment upregulated 1297 genes in the sensitive model: 1175 specific to the sensitive H358 cells after adagrasib treatment and 122 overlapping genes between H358 and H358A-R cells. Similarly, only 259 of the genes that were downregulated in the adagrasib-treated H358 cells were also expressed in the treated resistant cells, indicating fundamental differences in the response of sensitive and resistant cells to drug treatment (Fig. 4B).

To discern the comprehensive mechanisms driving drug resistance within our models, beyond individual genes, we evaluated the enriched transcriptomic signatures expressed under our experimental conditions, clustering pathways, and phenotypes. When comparing H358 sensitive cells before and after adagrasib treatment (Fig. 4C, top), adagrasib treatment visibly reduced KRAS and MTORC1 signaling but increased protein secretion.

We found that one of the most significant signatures relevant to the adagrasib-resistant phenotype was the epithelial-to-mesenchymal transition signature [27] which has been proposed as a mechanism for tumor cells to evade drug inhibition [22] (Fig. 4C and S4B). Acquired resistant cells also showed significantly increased expression of E2F and MYC targets (Fig. 4C, bottom). RT-PCR was used to validate the differences observed in the GSEA between the sensitive and adagrasib cells. The canonical epithelial marker E-cadherin (CDH1) was significantly reduced in H358A-R cells, in contrast to increased levels of mesenchymal N-cadherin (CDH2) and vimentin. POLE [28] and RAF [29], two E2F targets, were elevated in the adagrasib-resistant phenotype, as was RAN [30], a known MYC target (Fig. S4A).

Acquired drug resistance to KRAS^G12C^ inhibitors is likely a multifactorial process, in which several genes facilitate the alteration of gene expression. To identify potential therapeutic targets and drug candidates for targeting adagrasib-resistant cells, we examined our transcriptomic dataset in iLINCS, a platform that includes datasets and signatures of cellular perturbations [13]. We utilized transcriptomic analysis of H358 adagrasib-resistant cells to assess which chemicals or drugs could potentially reverse the adagrasib-resistant signature and their fundamental genes. The primary candidates identified in this analysis were SRC inhibitors, followed in decreasing order of significance by HSP90, MEK and PI3K inhibitors (Fig. 4E) and other the inhibitors that have already been suggested previously to synergize with KRAS^G12C^i like EGFR and AURK inhibitors [25,31]. Interestingly, SRC, MEK and EGFR are well-known HSP90 clients [32–34].

After careful evaluation of the top ten potential drug candidates for the treatment of adagrasib-resistant phenotypes, we classified the inhibitors into groups by target (Fig. S4C). Three of the ten top candidates for targeting drug resistance were HSP90 inhibitors. Other recognized drugs included RTK inhibitors (EGFR, IGF1R, and ERBB2), SRC, MEK, and PI3K inhibitors. Next, we performed a systematic analysis to highlight the commonalities between H358 sensitive cells after adagrasib treatment, which were also maintained after cells became fully resistant to adagrasib. Analyses showed that 5525 genes were upregulated in both cases. Given that several of these altered genes are involved in aberrant tumoral signal transduction, and many relevant oncogenic proteins are HSP90 clients, we compared the altered genes (FC > 2, FDR <0.05) with a curated list of known HSP90 clients. Remarkably, from the approximately 400 HSP90 clients, almost 30 % were increased in the adagrasib-tolerant cells and resistant cells that survived transient drug treatment (Fig. 4F and S4D).

### 3.5. Use of Hsp90 inhibitor with KRAS^G12C^ inhibitor to overcome drug resistance

**Fig. 5.**
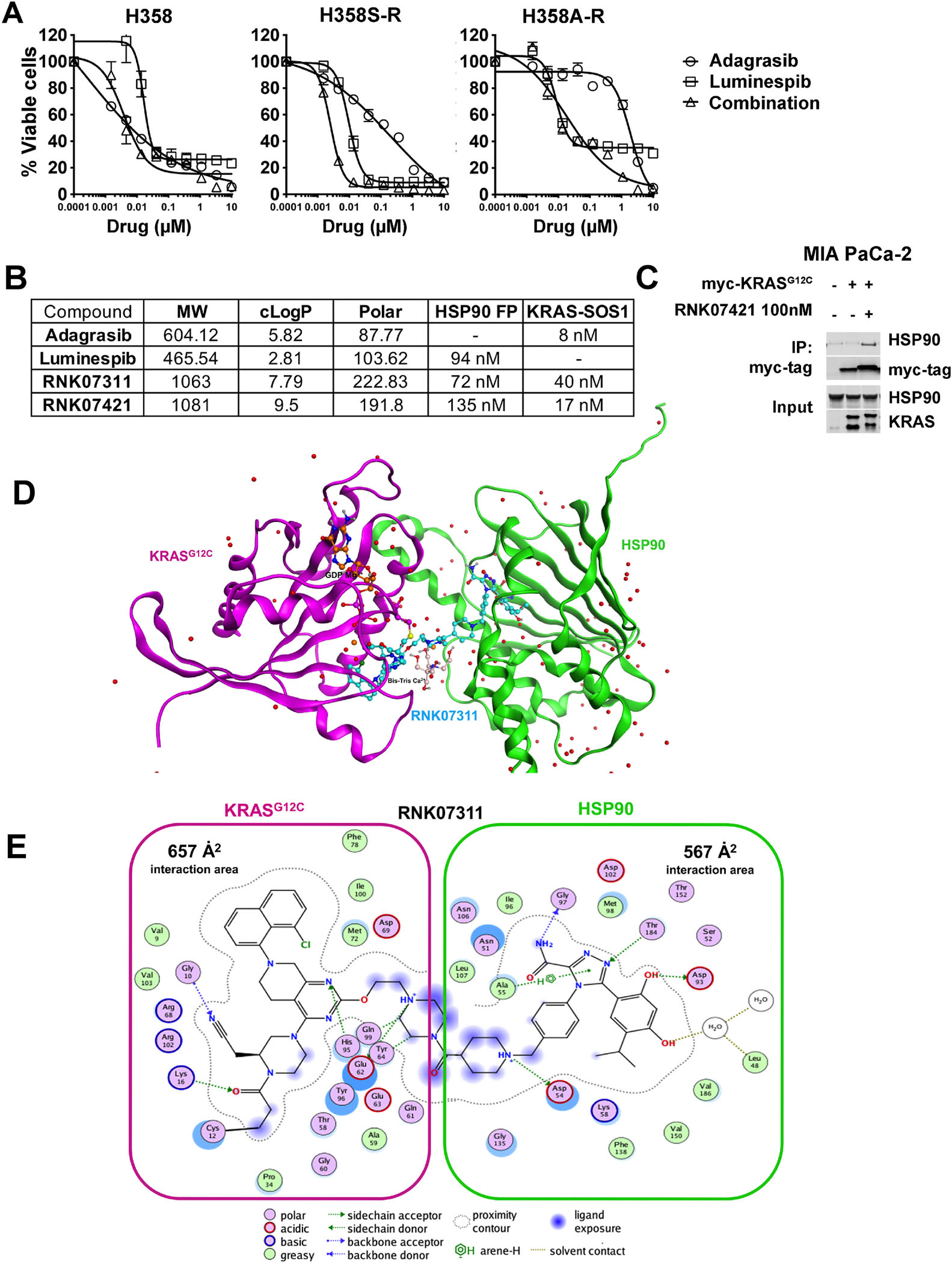
The combination of KRAS^G12C^ and HSP90 inhibitors can overcome drug resistance. (A) Colorimetric *in vitro* proliferation assays after combination treatment with adagrasib and HSP90 inhibitor, luminespib, for 72 h in each of the three H358 cell lines. Error bars: S.D. Representative image from two independent experiments. (B) Biochemical description of compounds: Adagrasib, luminespib, RNK07311 and RNK07421 (C) Immunoprecipitation of myc-KRAS^G12C^ transfected into MIA PaCa-2 cells treated with the heterobifunctional inhibitor, RNK07311 (100 nM), co-immunoprecipitates HSP90. (D) Crystal structure of KRAS (magenta) Cys12 covalently bonded to RNK07311 (cyan) while also binding the N-terminal ATP-pocket of HSP90 (light green). (Molecular water, GDP-Mg^2+^, and Bis-Tris-Ca^2+^ are also shown (PDB: RKCH). (E) Schematic of amino acids involved in KRAS^G12C^ and HSP90 binding to compound, RNK07311.

HSP90 is a molecular chaperone that stabilizes a wide variety of proteins, including RTKs, which are required for the survival of cancer cells [35–37]. There are numerous potential RTKs that can be activated to overcome drug inhibition; thus, we sought to assess the effect of combining Hsp90 inhibitors (luminespib) with KRAS G12C inhibitors in cell lines and PDOs. In adagrasib-sensitive cells, combination treatment provided a slight benefit, while resistant cells showed higher IC_50_ and reduced cell viability in the presence of adagrasib and luminespib (Fig. 5A and S5A). In PDOs, the combination of adagrasib and luminespib was the most efficient treatment to prevent reactivation of ERK, a surrogate marker for active proliferation and survival (Fig. S5B).

Advances in small molecule drug chemistry have led to the development of heterobifunctional compounds with two separate inhibitory activities and improved safety profiles [38,39]. Here, we show the structure and activity of two novel heterobifunctional agents capable of concomitantly targeting KRAS^G12C^ and inhibiting HSP90. RNK07421 and RNK07311 had nanomolar binding affinities for HSP90 and KRAS (Fig. 5B). In MIA PaCa-2 cells transfected with myc-KRAS^G12C^, RNK07421 treatment promoted the pulldown of HSP90 with immunoprecipitated myc-KRAS (Fig. 5C), and the crystal structure of KRAS^G12C^ covalently linked via Cys12 to RNK07311 binding to the N-terminus of human HSP90α. The 1.96 Ȧ structure depicts KRAS^G12C^ in the GDP-Mg^2+^ bound inactive state and contains a Ca2+ ion and Bis-Tris molecule coordinated between the RNK07311 linker and the two proteins (Fig. 5D). RNK07311 binds KRAS^G12C^ and HSP90, analogous to adagrasib and NVP-AUY922, respectively. The buried interaction surface area between KRAS^G12C^ and RNK07311 covers 657 Ȧ^2^ while the surface between HSP90 and RNK07311 covers 567 Ȧ^2^ (Fig. 5E). The protein-protein interaction interface includes seven KRAS^G12C^ residues and four HSP90 residues at a distance of 2.75 Ȧ including a salt-bridge between Glu91 of KRAS^G12C^ and Lys58 of HSP90. Another key interface involves His94 of KRAS^G12C^ and Tyr61 of HSP90, which is phosphorylated and may play a role in client-protein interactions [40]. A separate interface included Asp132, Leu133, and Ser136 of KRAS^G12C^, and Leu64 and Thr65 of HSP90 (Fig. S5C). This structure demonstrates how the compound RNK07311 induces a novel protein-protein interaction between KRAS^G12C^ and HSP90, which does not naturally occur in untreated cells.

The designed compounds exhibited a range of capacities to reduce pERK levels (Figs. S5D and S5D). Importantly, both RNK07421 and RNK07311 prevented pERK reactivation for 72 h in both the NSCLC H358 and PDAC MIA PaCa-2 cell lines (Figs. S5D and S5E). In dose-escalation experiments, RNK07421 demonstrated superior efficiency to RNK07311 in reducing pERK levels at lower drug concentrations than RNK07311 in the KRAS^G12C^ mutant tumor models (Figs. S5D and S5E). In addition, RNK07421 effectively reduced HSP90 client protein levels, including those of cRAF and CDK4 (Figs. S5D and S5E). In NSCLC sotorasib- and adagrasib-resistant cells, treatment with RNK07421 significantly reduced cell viability, suggesting that RNK07421 has the potential to overcome acquired drug resistance to KRAS^G12C^ inhibitors (Fig. S5F).

### 3.6. RNK07421 heterobifunctional inhibitor of KRAS^G12C^ and HSP90 can overcome innate and acquired drug resistance to KRAS^G12C^ inhibitors

**Fig. 6.**
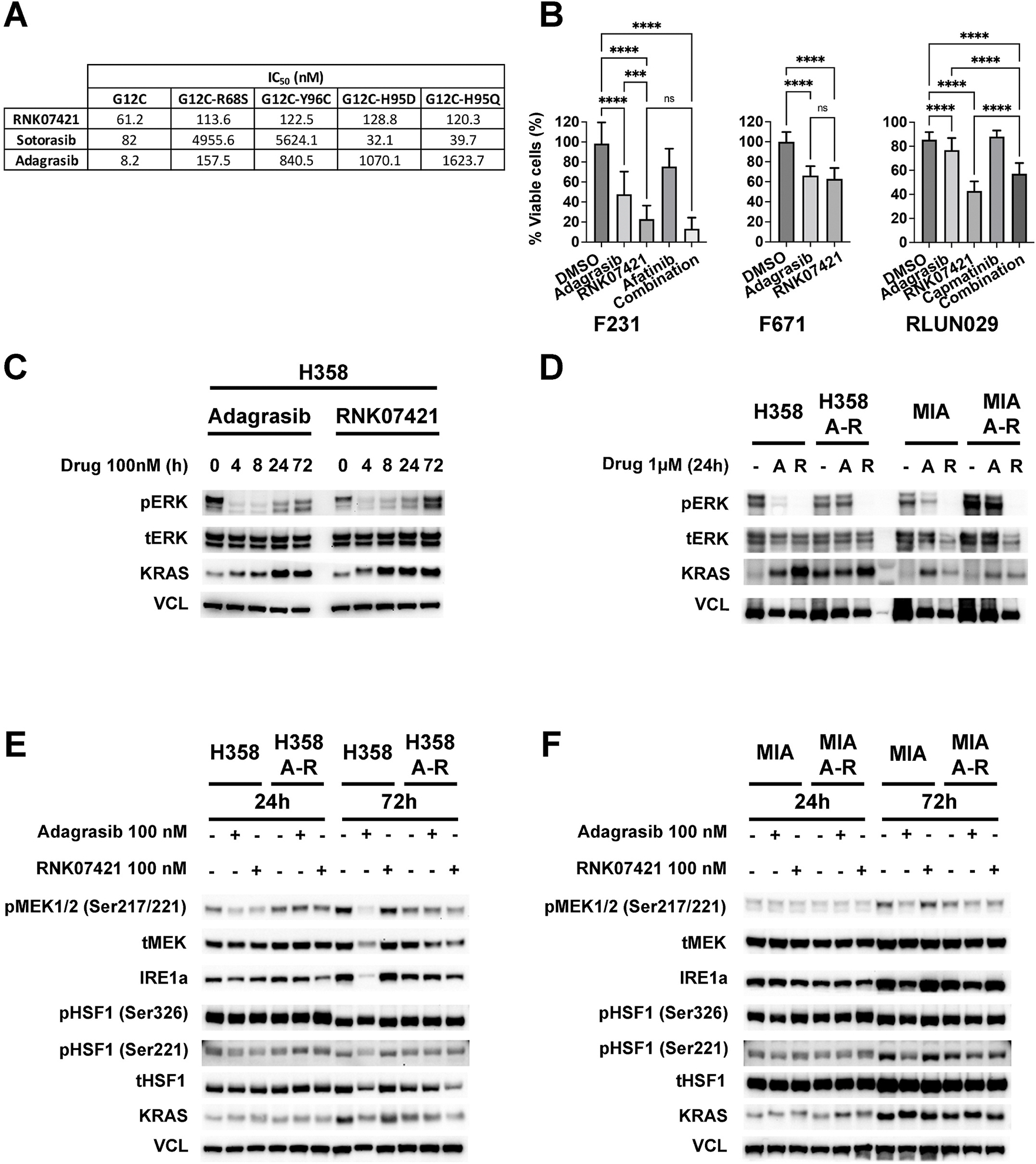
RNK07421 heterobifunctional agent can overcome innate and acquired drug resistance to KRAS^G12C^ inhibitors. (A) IC_50_ values (nM) of Ba/F3 cells carrying the annotated mutations and treated with RNK07421, sotorasib or adagrasib. CellTiter-Glo^®^ (Promega) was used to determine cell viability. (B) Percent of viable F231, F671 and RLUN029 PDOs after 72 h of treatment with the indicated inhibitor or inhibitor combination including vehicle treatment (DMSO) as a control. Average of three independent experiments. Error bars: S.D; One-way ANOVA. ****P < 0.0001. (C) Immunoblots of H358 cells after treatment with adagrasib or RNK07421 for varying timepoints between 0 and 72 h. Representative immunoblots from two independent experiments are shown. Vinculin (VCL) as a loading control. (D) Immunoblots comparing H358 and MIA PaCa-2 sensitive and adagrasib resistant cells after adagrasib or RNK07421 treatment for 24 h. Representative immunoblots from two independent experiments are shown. Vinculin (VCL) as a loading control. (E) Immunoblots comparing H358 sensitive cells to H358 A-R cells after adagrasib or RNK07421 treatment for 24 or 72 h. Experiments were repeated and blots made with MIA PaCa-2 and MIA PaCa-2A-R cells. Representative immunoblots from two independent experiments are shown. Vinculin (VCL) as a loading control.

The acquisition of new secondary mutations in KRAS has been shown to bypass KRAS^G12C^ inhibition and promote drug resistance [6,23,24] prompting the evaluation of the potential of RNK07421 against secondary mutations. KRAS^G12C^ dependent mouse Ba/F3 cells harboring secondary mutations (R68S, Y96C, H95D, and H95Q), known to confer resistance to KRAS^G12C^ inhibitors, were challenged with RNK07421, sotorasib, or adagrasib. In all secondary mutations evaluated, RNK07421 provided a favorable IC_50_ profile compared to either sotorasib or adagrasib (Fig. 6A). Furthermore, RNK07421 reduced the viability of F231, F671, and RLUN029 PDOs regardless of their sensitivity to adagrasib (Fig. 6B and S6A). Previous results suggested that the combination of adagrasib with afatinib or capmatinib may produce a synergistic effect in F231 and RLUN029 cells (Fig. S3E). Interestingly, RNK07421 caused a greater reduction in cell viability than the two-drug combination in both PDOs (Fig. 6A and S6A). The compromised viability correlated with a marked reduction in ERK phosphorylation (Fig. S6B). Furthermore, RNK07421 selectively targeted all PDOs regardless of its sensitivity to adagrasib (Fig. 3B and S3C). RNK07421 further reduced pERK levels when compared to adagrasib for up to 72 h post treatment (Fig. 6C). Additional evaluation of the new compound showed that RNK07421 efficiently suppressed ERK phosphorylation in both sensitive and adagrasib-resistant H358 and MIA PaCa-2 cells (Fig. 6D).

Modulation of proteostasis, the ability to overcome imbalanced protein homeostasis, has been shown to contribute to drug resistance to KRAS^G12C^ inhibitors [9]. We investigated whether RNK07421 affects the molecular network responsible for maintaining protein balance. Since proper protein synthesis and folding are crucial for cancer cell survival, we examined whether RNK07421 treatment disrupted these processes. The activation of HSF1 and IRE1a, which are involved in detecting protein errors and maintaining protein quality control in the cytosol and ER, respectively, was evaluated [41–43]. RNK07421 did not noticeably alter HSF1 or IRE1α protein levels or activation. Adagrasib affected HSF1 activation and IRE1α levels in parental cell lines but had no effect on the evolved adagrasib-resistant lines (Fig. 6E–F). These observations do not prove, but suggest, that acute disruption of proteostasis is not relevant in the acquisition of resistance in our NSCLC and PDAC models.

### 3.7. In vivo activity of the heterobifunctional HSP90 & KRAS^G12C^ inhibitor

**Fig. 7.**
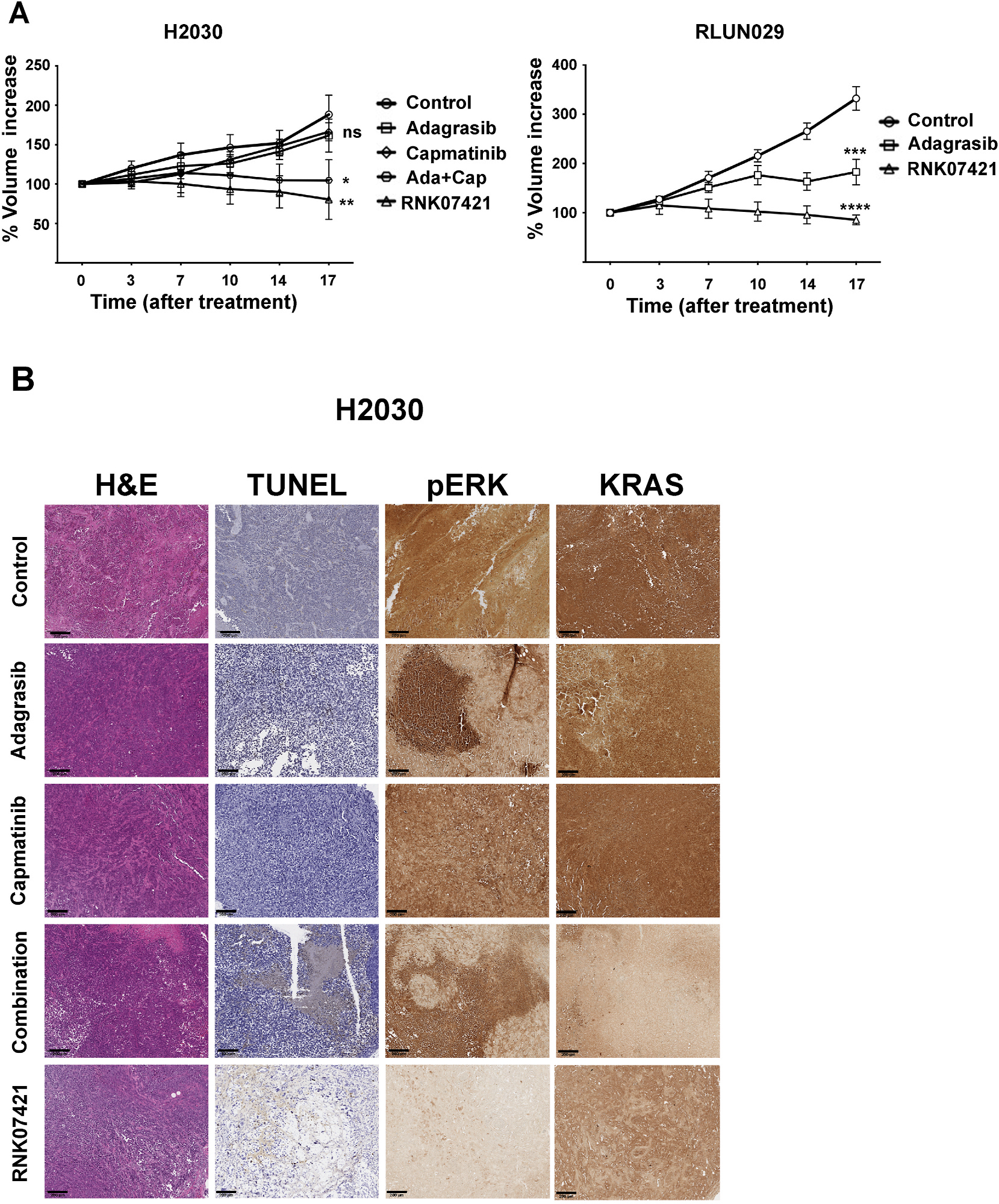
*In vivo* dual inhibition of KRAS and HSP90 is more effective than adagrasib alone. (A) % of average mouse tumor volumes in H2030 and RLUN029 xenograft mice measured for up to 17 days post-treatment. Treatments included vehicle, adagrasib (50 mg/kg), capmatinib (10 mg/kg), combination adagrasib and capmatinib (100 and 10 mg/kg respectively), or RNK07421 (100 mg/kg). Error bars: SEM, One-way ANOVA. Not significant, *P < 0.05, **P < 0.01, ***P < 0.001, ****P < 0.0001. (B) Representative images of tumor tissue from H2030 xenograft mice stained with H&E, TUNEL, or immunohistochemically with antibodies for pERK and KRAS respectively.

We next tested RNK07421 as compared to several drug treatments in innate resistant H2030 and RLUN029 PDO xenografts to evaluate their potential *in vivo* activity. Given that H2030 tumor cells can evade adagrasib inhibition and maintain elevated levels of MET, as shown previously (Fig. 3E–F), we included treatment with capmatinib, a MET inhibitor, in our experiments. RNK07421-treated tumors showed a significant reduction in volume compared to controls (351 ± 112 mm^3^ vs. 146 ± 43 mm^3^, control vs. RNK07421); however, tumors treated with a combination of adagrasib and capmatinib were significantly smaller than the RNK07421-treated tumors (185 ± 41 mm^3^ vs. 146 ± 43 mm^3^, combination vs. RNK07421; Fig. 7A left). H2030 xenografts did not respond to adagrasib treatment (351 ± 112 mm^3^ vs. 236 ± 40 mm^3^, control vs. adagrasib), consistent with the capmatinib-treated tumors (Fig. 7A left). RLUN029 tumors exhibited a partial response to adagrasib treatment, as indicated by a statistically significant difference in tumor volume compared to the vehicle-treated group (Fig. 7A).

To further assess the effect of the drug *in vivo*, mouse body weight was measured three times per week to highlight the criteria for tolerated drug dosing. Overall, the drug-treated mice maintained or increased their body weight over time, except for RNK07421-treated mice, which lost approximately 15 % of their total body weight by the end of the designated treatment period (Fig. S7A).

After confirming treatment-induced cell death in the tumors by TUNEL staining, adagrasib treatment resulted in minimal cell death in both xenograft models, especially when compared to RNK07421-treated tumors (Fig. 7B). In H2030 tumor xenografts, the combination of adagrasib, METi, and capmatinib resulted in elevated levels of cell death. Immunohistochemical staining indicated that dual inhibition of KRAS and HSP90 with RNK07421 reduced pERK, a surrogate for KRAS activation, consistent with the KRAS antibody marker (Fig. 7B). These results are consistent with those of the RLUN029 strains (Fig. S7B).

## 4. Discussion

KRAS was previously deemed “undruggable” until advancements in biomolecular chemistry in the last decade [44,45] which led to a fundamental shift in the treatment of KRAS-mutant tumors. FDA approval of two specific KRAS^G12C^ inhibitors, sotorasib and adagrasib, has greatly improved the prognosis of NSCLC patients harboring a common cysteine to glycine point mutation in codon 12 [1,2]. However, cases of both acquired and innate resistance to these inhibitors have been reported [23,24,46] and need to be addressed.

Although both sotorasib and adagrasib are highly selective, potent, and bind specifically to inactive (GDP-state) KRAS^G12C^, our results show considerable differences between adagrasib- and sotorasib-resistant cell lines. Our results indicated that sotorasib-resistant cells remained sensitive to adagrasib, suggesting that sotorasib-resistant cells may have some level of continued dependence on KRAS, unlike adagrasib-resistant cells that were fully resistant to both sotorasib and adagrasib (Fig. 1A and S1A). Further studies should be performed to test the feasibility of treating sotorasib-resistant tumors with adagrasib as second-line FDA-approved treatment.

Unfortunately, acquired resistance to adagrasib remains a significant therapeutic challenge. RTK activation in our adagrasib-resistant models was lineage-specific (Fig. 3B and D, S3C, and S3D), highlighting the importance of developing proper combination therapies. However, when designing potential combination therapies, it is important to be aware of the potential toxicities associated with drug combinations. This may hinder the translational capacity of the combination therapies.

A common challenge often encountered in clinical settings is the broad spectrum of drug sensitivity observed among patients with the same oncogenic mutation. This range of drug sensitivities may be related to oncogenic dependence, which is consistent with our models that show that tumors are addicted to aberrant proliferating signals triggered by KRAS (Fig. 1E–S1E, S3B). The correlation between low copy number of oncogenic driver mutations in KRAS and discernible drug resistance is consistent with prior research [47,48]. In three of the different NSCLC PDOs evaluated, RLUN029 showed the most significant resistance to both sotorasib and adagrasib (Fig. 3B&S3C), while presenting low copy numbers of the KRAS^G12C^ gene (Fig. S3B). Taken together, these results suggest that tumors may have reduced dependency on KRAS signaling despite the presence of oncogenic driving mutations.

Resistance to G12C inhibitors is comprised of multiple layers of complexity, from the acquisition of secondary mutations in oncogenes, such as KRAS, to the activation of parallel signaling pathways that sustain tumor proliferation. Numerous mutations in KRAS have been described as drivers of drug resistance [5,24]; however, analysis of our panel of sotorasib- and adagrasib-resistant cell lines and PDOs did not highlight any of the previously described secondary mutations (Fig. 1F–S1F, and S4A). However, we detected an unexpected mutation, glycine to arginine at codon 114 (G114R), in two of our unique inhibitor-resistant tumor models (lung and pancreatic tumor cell lines). Further clarification is required to determine the significance of this mutation, particularly considering its heterozygous nature and location of this mutation within the catalytic domain [49].

The reduced dependency on oncogenic KRAS might be counterbalanced by the feedback activation of mediators within the RTK-KRAS-MAPK proliferation cascade [7]. While innate resistance models predominantly activated a small number of RTKs to escape drug inhibition (Fig. 3E), the acquisition of drug resistance led to visible activation of virtually all the RTKs evaluated. This scenario does not encourage the design of effective combination treatments given the multitude of concomitantly activated proliferative and survival signals. Ideally, combination treatments should be designed to be applicable to large subsets of patients. Co-targeting of EGFR and KRAS^G12C^ demonstrates enhanced drug sensitivity *in vitro* and has been proven effective in clinical trials [2,50,51].

Transcriptomic analysis of our cell models allowed us to distinguish between extraneous adaptive mechanisms of survival and the highly relevant mechanisms that underlie drug resistance (Fig. 4A and B) [52, 53]. For example, adagrasib-resistant cells have been shown to be enriched in signatures that sustain transcriptomic activity and receptor tyrosine regulation, suggesting that drug treatment can drive cells to highly active phenotypes to escape oncogenic inhibition. The complexity of adagrasib resistance suggests that sufficient pharmacological inhibition will require the RAS pathway to be targeted at multiple levels. KRAS^G12C^ inhibitor combination treatments that exhibit pleiotropic effects will likely induce suppression of oncogenic KRAS and downstream nodes as a means for tumor cells to maintain survival. According to our analysis of the pharmacological vulnerabilities associated with resistance phenotypes, HSP90 inhibitors are the leading contenders for sensitizing cells that have developed acquired resistance (Fig. 4E).

Pimitespib (TAS-116) is an oral HSP90 inhibitor approved in Japan for the treatment of patients with GIST that has progressed to chemotherapy. The drug is currently being evaluated in clinical trials to determine its safety and tolerability in solid tumors [54]. Given the preliminary success of this drug, we designed a novel compound, RNK07421, to heterobifunctionally block KRAS^G12C^ and inhibit HSP90 activity simultaneously. We showed that this dual activity was effective in drug-sensitive, but more importantly, drug-resistant, cell lines (Fig. 6D) and PDOs (Fig. 6B & S6B). Moreover, this heterobifunctional molecule could have additional effects due to the inhibition of HSP90 functions because it could lead to wrong proteins, such as the KRAS adducts formed, to the E3 ligase pathways for degradation.

Heterobifunctional agents offer several advantages over combination treatments, minimizing dose-limiting toxicities while maximizing therapeutic effects [55]. Trastuzumab deruxtecan is an antibody-drug conjugate that has been approved for the treatment of HER2 positive NSCLC patients [56] and shows promising outcomes in cancer treatment. In this case, the main oncogenic driver, KRAS, can be completely inhibited in sensitive cells, while the drivers of drug resistance will be continually targeted. Interestingly, the specificity conferred by the adagrasib warhead in RNK07311 and RNK07421 may help achieve the therapeutic window while simultaneously inhibiting HSP90, thus performing the duty of combination treatments with a single drug [17,57–60]. Moreover, HSP90 inhibitors accumulate in proliferating tumor cells [61,62]. Heterobifunctional molecules, such as RNK07421 and RNK07311, also offer a promising approach to ameliorate the difficulties of combining multiple inhibitors with different pharmacodynamic and pharmacokinetic profiles. The KRAS^G12C^i moiety confers specificity to these molecules while the HSP90i can target several HSP90 clients relevant in the drug resistance phenotype. In addition, the dosing -relevant to prevent the increase of some HSP90 clients [16]-can be better controlled with dual molecules. Single heterobifunctional molecule treatments reduce toxicity and side effects and simplify dosing regimens for patients.

Our data showed that, under drug pressure, tumor cells promoted the expression of several MAPK pathway elements (Fig. 2C–S3E, 4C) that require HSP90 activity to ensure proper folding, protein function, cell survival, and tumor maintenance. The central role of HSP90 in these processes can be exploited to block multiple targets using the same inhibitor. Due to the vast number of HSP90 clients available, a broader set of patients may benefit from this dual-function approach, potentially reducing the need for prior stratification. The dual activity of RNK07421 will likely benefit both KRAS^G12C^i responders and non-responders by adding HSP90 activity to the treatment and improving the therapeutic index in highly heterogeneous tumors. Moreover, this single molecule has the potential to reduce the number of medications taken by patients, consequently limiting the probable side effects and toxicities associated with combining medications [63–65].

## Supplementary Material

1

2

## Data Availability

Research data supporting this publication will be available upon acceptance.

## Appendix A. Supplementary data

Supplementary data to this article can be found online at https://doi.org/10.1016/j.canlet.2025.217691.
