# Supplementary material for: Chaperone directed heterobifunctional molecules circumvent KRASG12C inhibitor resistance
Source: Cancer Lett. Author manuscript; Available in PMC 2026 Jun 18. (PMC13276862; doi:10.1016/j.canlet.2025.217691)
Supplement: 1 [file NIHMS2173876-supplement-1.pdf]

Figure S1

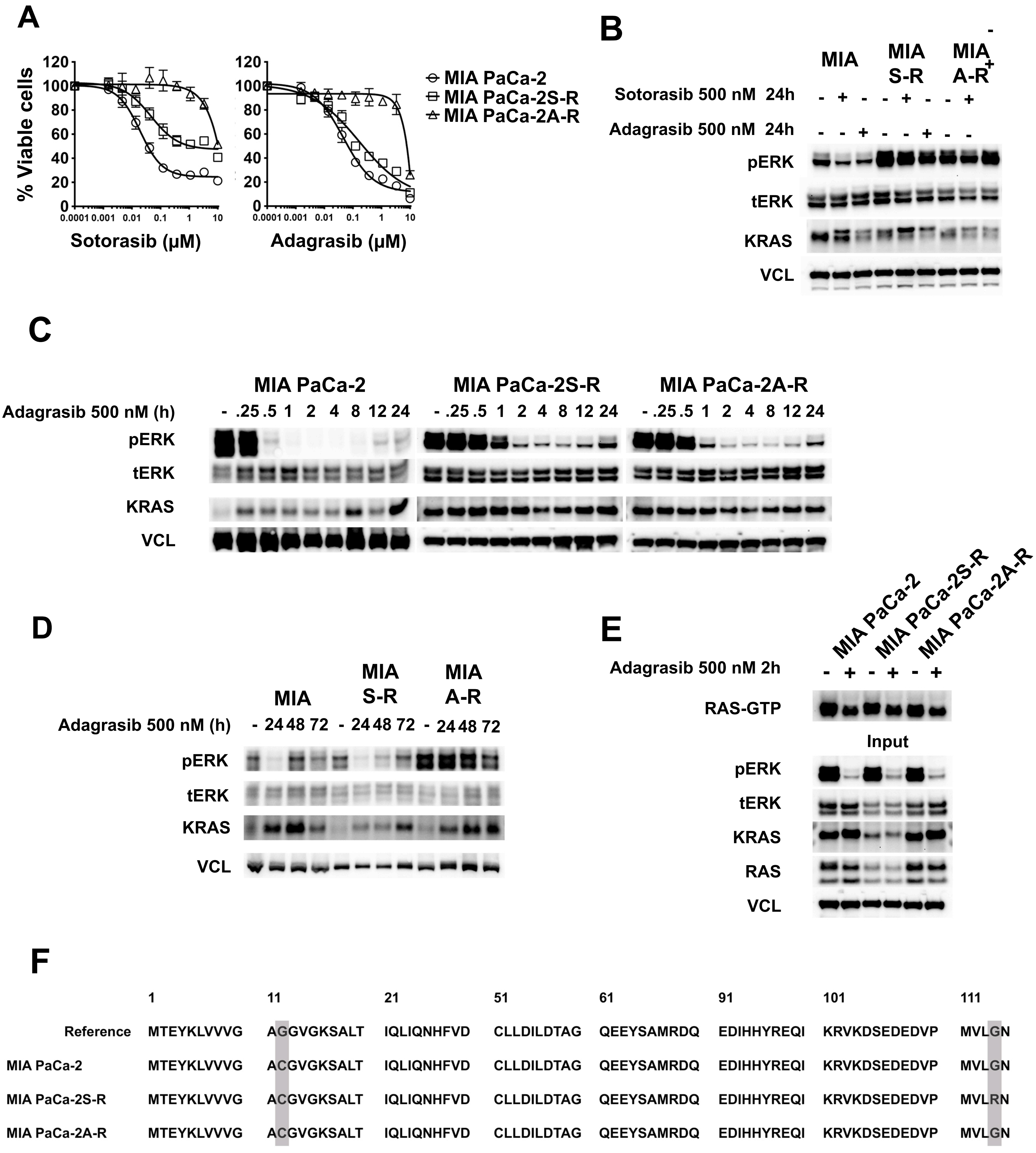

A
% Viable cells
120
100
80
60
40
20
0
0.0001 0.001 0.01 0.1 1 10
Sotorasib (μM)
Adagrasib (μM)
MIA PaCa-2
MIA PaCa-2S-R
MIA PaCa-2A-R
B
MIA
MIA S-R
MIA A-R
Sotorasib 500 nM 24h
Adagrasib 500 nM 24h
pERK
tERK
KRAS
VCL
C
MIA PaCa-2
MIA PaCa-2S-R
MIA PaCa-2A-R
Adagrasib 500 nM (h)
- .25 .5 1 2 4 8 12 24
pERK
tERK
KRAS
VCL
D
MIA
MIA S-R
MIA A-R
Adagrasib 500 nM (h)
- 24 48 72
pERK
tERK
KRAS
VCL
E
MIA PaCa-2
MIA PaCa-2S-R
MIA PaCa-2A-R
Adagrasib 500 nM 2h
RAS-GTP
Input
pERK
tERK
KRAS
RAS
VCL
F
1 11 21 51 61 91 101 111
Reference MTEYKLVVVG AGGVGKSALT IQLIQNHFVD CLLDILDTAG QEEYSAMRDQ EDIHHYREQI KRVKDSEDEDVP MVLGN
MIA PaCa-2 MTEYKLVVVG ACGVGKSALT IQLIQNHFVD CLLDILDTAG QEEYSAMRDQ EDIHHYREQI KRVKDSEDEDVP MVLGN
MIA PaCa-2S-R MTEYKLVVVG ACGVGKSALT IQLIQNHFVD CLLDILDTAG QEEYSAMRDQ EDIHHYREQI KRVKDSEDEDVP MVLRN
MIA PaCa-2A-R MTEYKLVVVG ACGVGKSALT IQLIQNHFVD CLLDILDTAG QEEYSAMRDQ EDIHHYREQI KRVKDSEDEDVP MVLGN

Figure S2

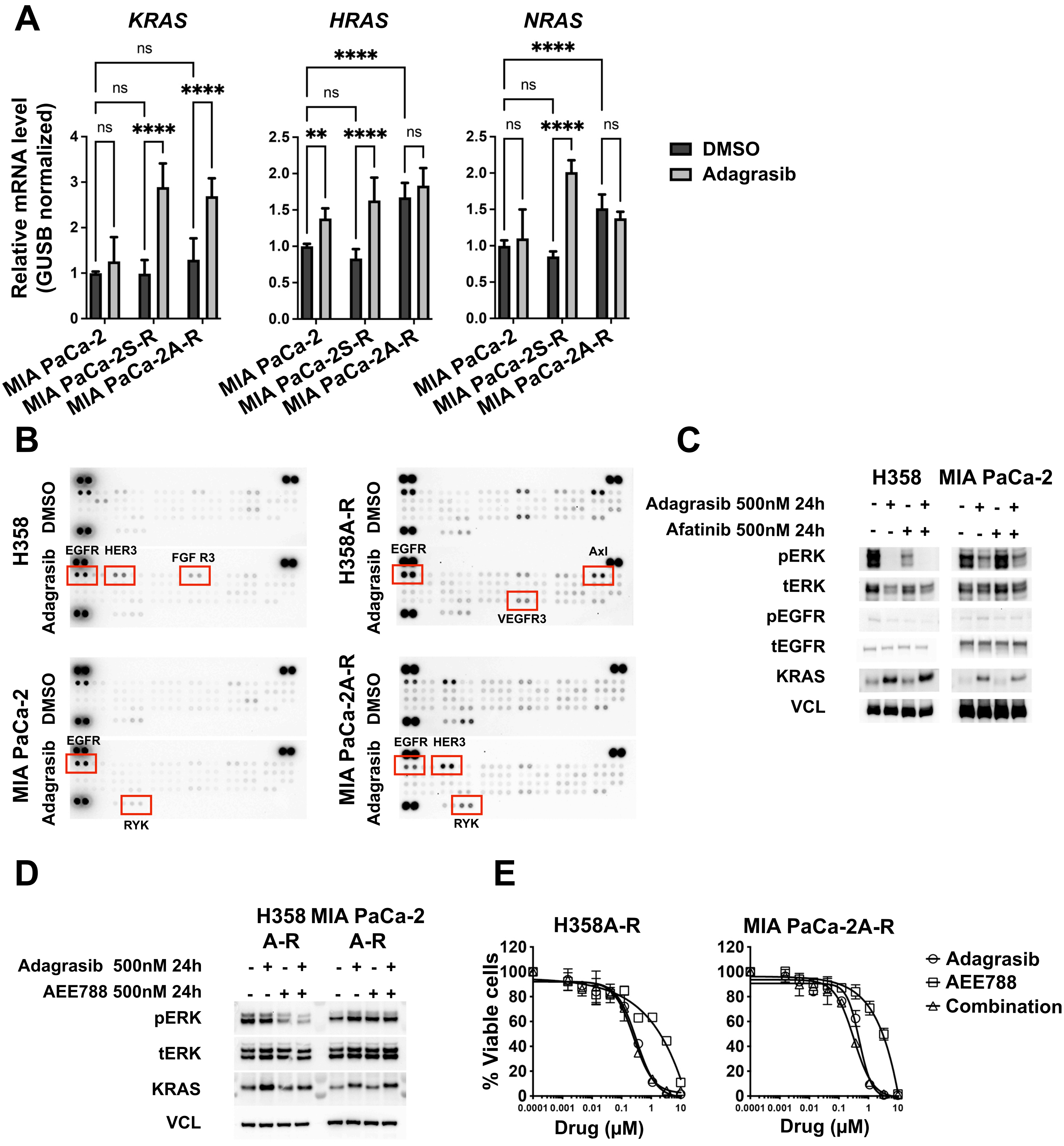

A
KRAS
HRAS
NRAS
Relative mRNA level (GUSB normalized)
ns
****
**
DMSO
Adagrasib
MIA PaCa-2
MIA PaCa-2S-R
MIA PaCa-2A-R
B
H358
H358A-R
MIA PaCa-2
MIA PaCa-2A-R
DMSO
Adagrasib
EGFR
HER3
FGF R3
VEGFR3
Axl
RYK
C
H358
MIA PaCa-2
Adagrasib 500nM 24h
Afatinib 500nM 24h
pERK
tERK
pEGFR
tEGFR
KRAS
VCL
D
H358 A-R
MIA PaCa-2 A-R
Adagrasib 500nM 24h
AEE788 500nM 24h
pERK
tERK
KRAS
VCL
E
H358A-R
MIA PaCa-2A-R
% Viable cells
Drug (μM)
Adagrasib
AEE788
Combination

Figure S3

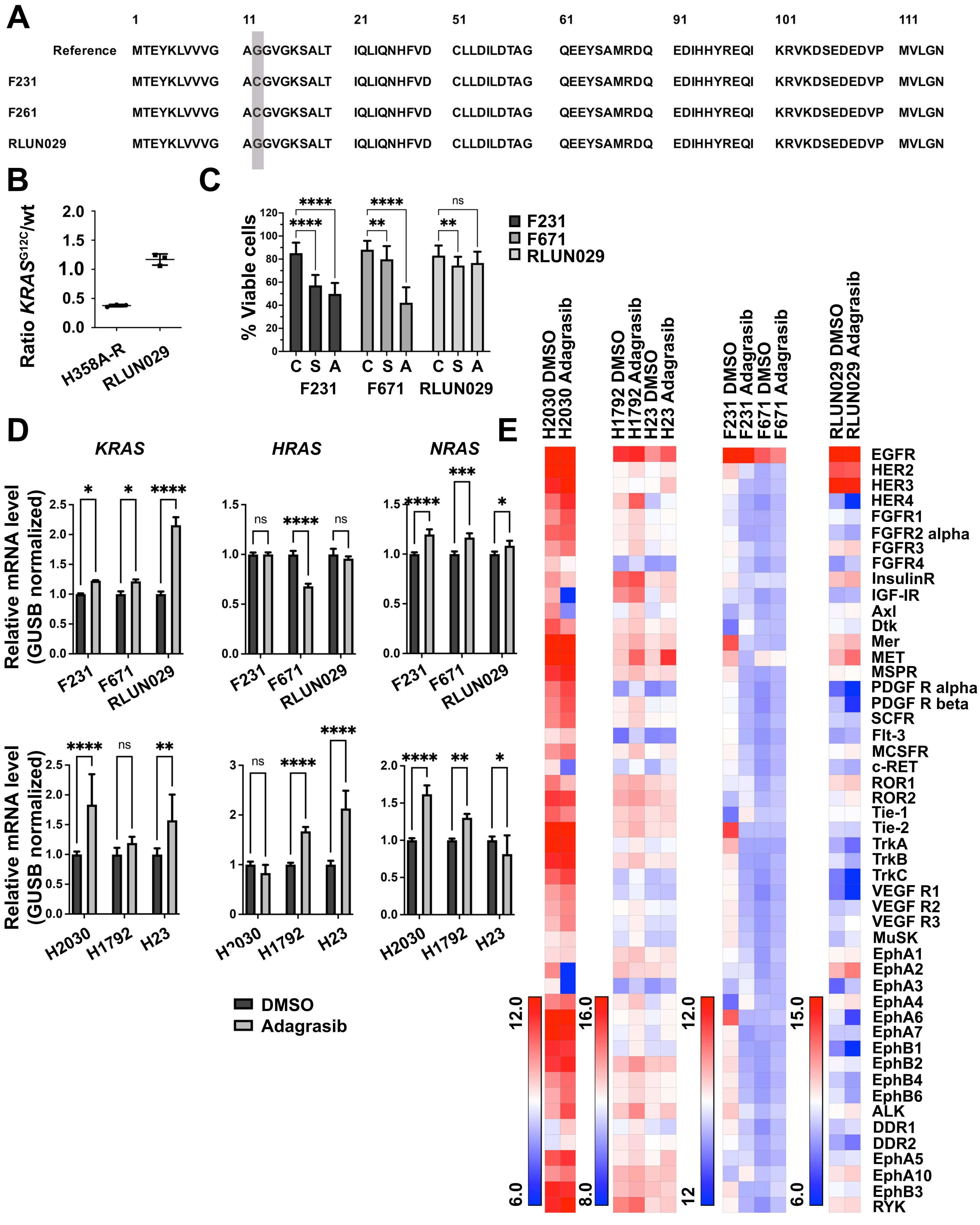
A
1 11 21 51 61 91 101 111
Reference MTEYKLVVVG AGGVGKSALT IQLIQNHFVD CLLDILDTAG QEEYSAMRDQ EDIHHYREQI KRVKDSEDEDVP MVLGN
F231 MTEYKLVVVG ACGVGKSALT IQLIQNHFVD CLLDILDTAG QEEYSAMRDQ EDIHHYREQI KRVKDSEDEDVP MVLGN
F261 MTEYKLVVVG ACGVGKSALT IQLIQNHFVD CLLDILDTAG QEEYSAMRDQ EDIHHYREQI KRVKDSEDEDVP MVLGN
RLUN029 MTEYKLVVVG AGGVGKSALT IQLIQNHFVD CLLDILDTAG QEEYSAMRDQ EDIHHYREQI KRVKDSEDEDVP MVLGN
B
Ratio KRAS G12C/wt
2.0 1.5 1.0 0.5 0.0
H358A-R
RLUN029
C
% Viable cells
120 100 80 60 40 20 0
**** **** ** **** ** ns
C S A C S A C S A
F231 F671 RLUN029
F231
F671
RLUN029
D
KRAS
HRAS
NRAS
Relative mRNA level (GUSB normalized)
* * **** ns **** ns **** *** *
F231 F671 RLUN029
**** ns ** ns **** **** **** ** *
H2030 H1792 H23
DMSO
Adagrasib
E
H2030 DMSO
H2030 Adagrasib
H1792 DMSO
H1792 Adagrasib
H23 DMSO
H23 Adagrasib
F231 DMSO
F231 Adagrasib
F671 DMSO
F671 Adagrasib
RLUN029 DMSO
RLUN029 Adagrasib
EGFR
HER2
HER3
HER4
FGFR1
FGFR2 alpha
FGFR3
FGFR4
InsulinR
IGF-IR
Axl
Dtk
Mer
MET
MSPR
PDGF R alpha
PDGF R beta
SCFR
Flt-3
MCSFR
c-RET
ROR1
ROR2
Tie-1
Tie-2
TrkA
TrkB
TrkC
VEGF R1
VEGF R2
VEGF R3
MuSK
EphA1
EphA2
EphA3
EphA4
EphA6
EphA7
EphB1
EphB2
EphB4
EphB6
ALK
DDR1
DDR2
EphA5
EphA10
EphB3
RYK
12.0 6.0
16.0 8.0
12.0 12
15.0 6.0

Figure S4

A

H358 DMSO vs MRTX

	FDR q value	NES
KRAS Signaling Down	<0.0	-1.642651
mTORC signaling	<0.0	1.910841
Protein secretion	0.074	-1.478753

H358 vs H358A-R

	FDR q value	NES
Epithelial and Mesenchymal Transition	<0.0	-2.134242
E2F Targets	<0.0	-2.07822
MYC Targets	<0.0	-2.000791

B

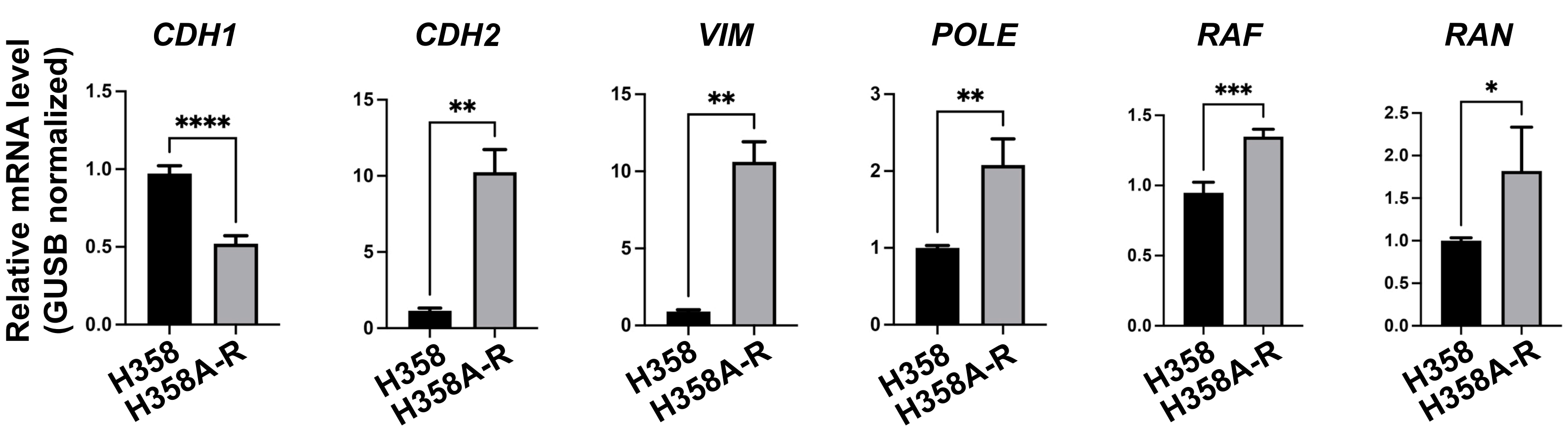

C

Perturbagen	Target	zScore	LOG10 (Pvalue)
Dasatinib	SRC	14.5	-47.3
13-Hydroxy-8,14,19-trimethoxy-4,10,12,16-tetramethyl-3,20,22-trioxo-2-azabicyclo[16.3.1]docosa-1(21),4,6,10,18-pentaen-9-yl carbamate	HSP90	13	-38.5
PD-0325901	MEK	11.9	-32.5
1166227-08-2	PI3K	10.4	-24.7
SCHEMBL16226502	HSP90	9.2	-19.9
Saracatinib	SRC	9	-19
GSK 1059615	PI3K	8.6	-17.5
NVP-AUY922	HSP90	8.6	-17.4
Pelitinib	EGFR	7.8	-14.4
Selumetinb	MEK	7.6	-13.9

D

HSP90&H358vsH358A-R&H358 C vs H358 Adagrasib										
RUNX1T1	INSR	ERBB3	GBP1	WASL	THRB	GABARAPL2	DARS	PRKCE	BRCA2	EPHA2
FN1	FGFR3	AHR	PRKCD	HTT	MAP3K1	CDK4	PTGES3	FHIT	PFAS	HIF1A
NOS3	GABARAPL1	CPEB3	KEAP1	THRA	IGF1R	RHOBTB2	MB	NR1D1	SRPK1	FKBP5
TIMP2	GBA	DDR1	HSPB1	MAP3K3	NFKB2	NMNAT2	SMYD2	LOXL2	CDC25A	FGFR4
PPARA	APAF1	FNIP1	MAP3K11	STAT1	PRPF8	SMYD3	CASP8	STK33	ERN1	PLK3
RAB11FIP1	RIPK1	TRADD	APOBEC3C	APP	CRNKL1	PLCG2	TXN	F2R	SLC2A4	ITGA2
RHOB	PPARD	KDR	NR2C2	RALBP1	ARRB1	RANBP9	VDR	HSPD1	MET	TTK
DAPK1	PIK3AP1	NCSTN	CAPN1	TGFBR1	KDM4B	PARK7	RAB3A	CTNNB1	FAM162A	AKR1B10
CFLAR	SYK	CDH1	POLR2A	TSC2	TNFRSF1A	LMNA	UCHL1	FYN	PUS7	IL1B
IDH1	MLLT3	STARD13	CPEB2	NFKBIE	NBN	GDI1	PLAU	CAMKMT	TERT	CDC25C
FNIP2	BCL6	LMTK3	TJP1	SCAP	NFKB1	PTK6	CD38	MAP2K1	CEBPB	LCK
RIPK3	MUC1	MAP3K8	RAF1	SRC	TGFBR2	AXIN1	GRPEL1	RUVBL2	MST1R	

Figure S5

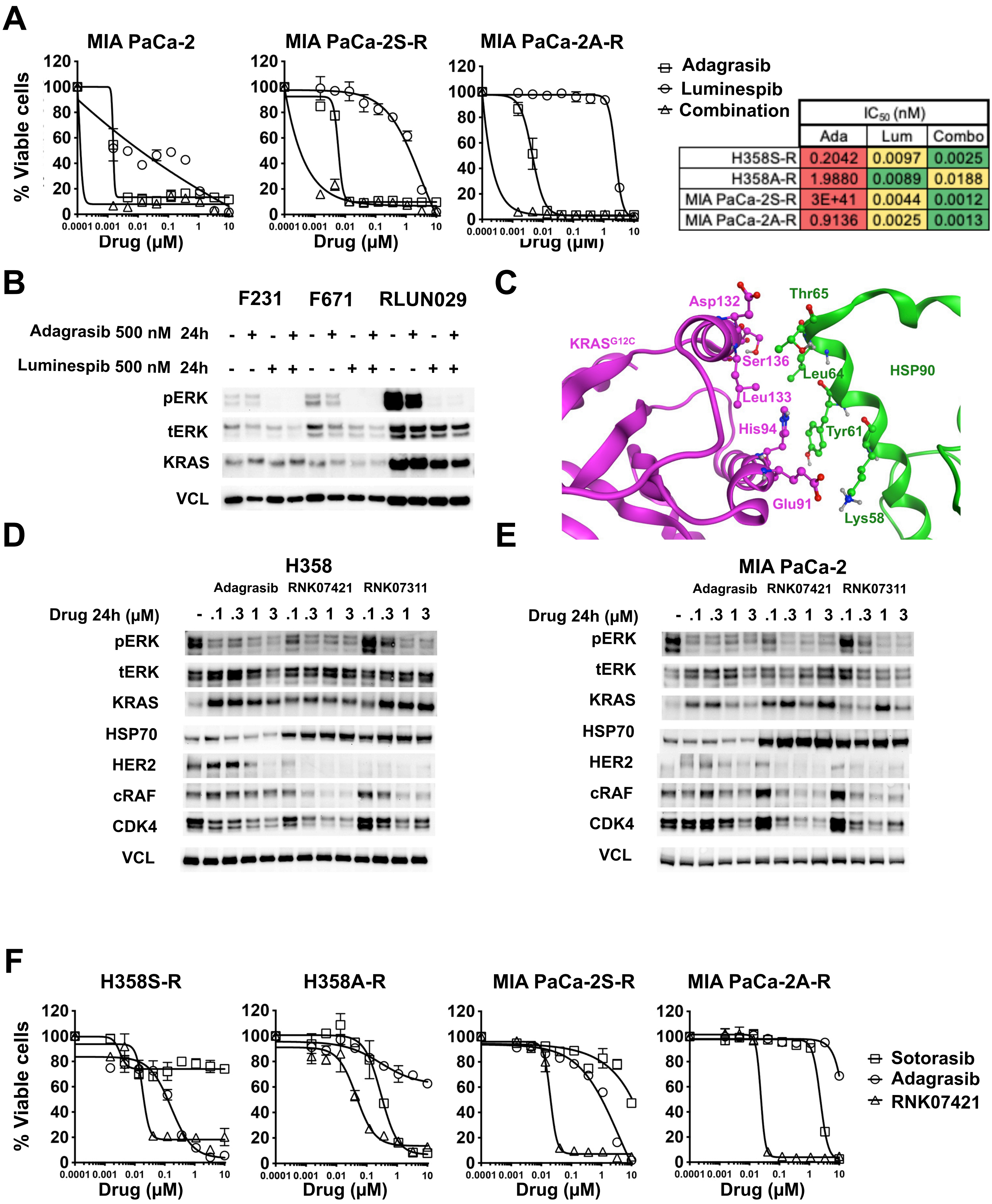

	IC_{50} (nM)		
	Ada	Lum	Combo
H358S-R	0.2042	0.0097	0.0025
H358A-R	1.9880	0.0089	0.0188
MIA PaCa-2S-R	3E+41	0.0044	0.0012
MIA PaCa-2A-R	0.9136	0.0025	0.0013

Figure S6

A

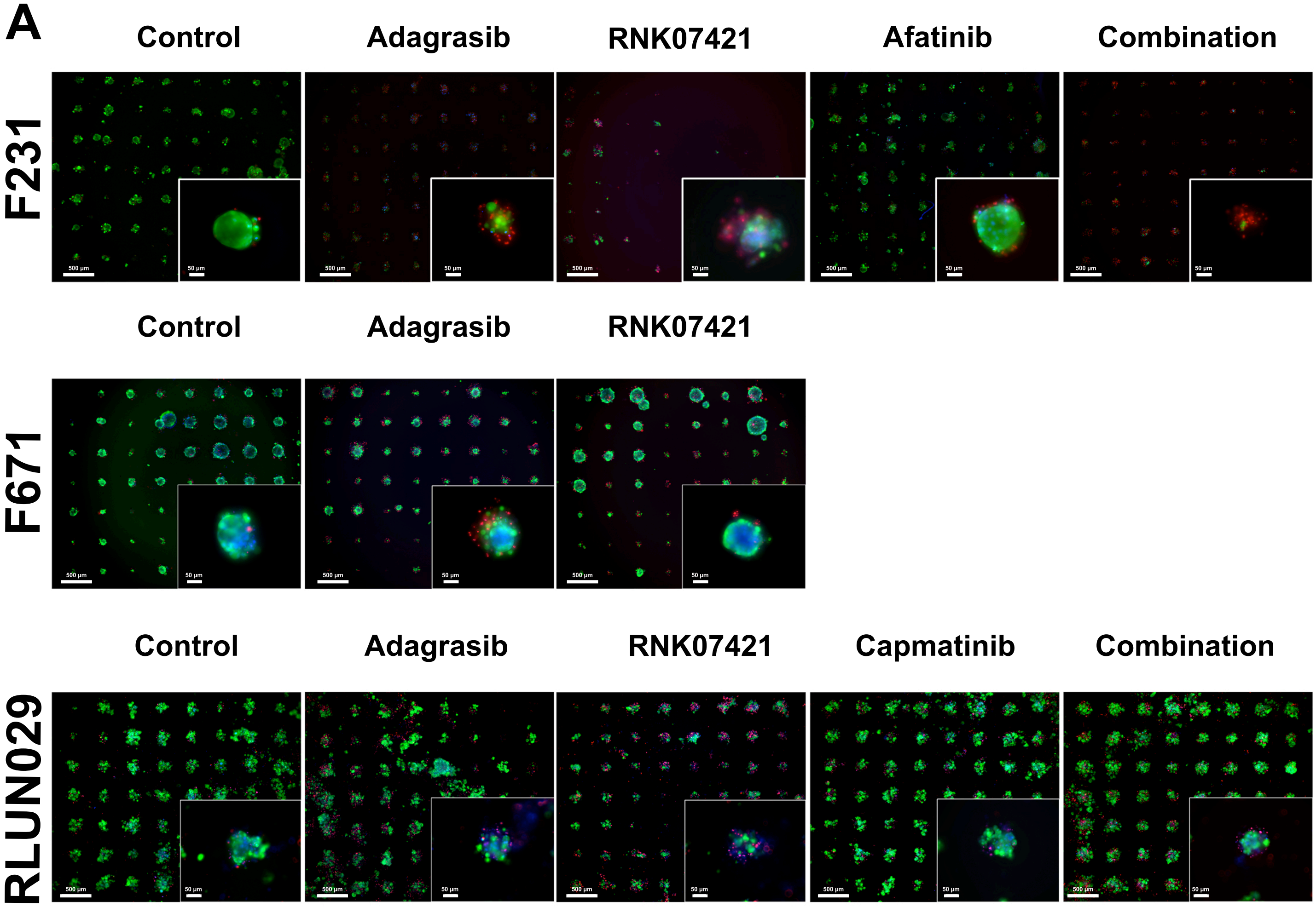
Control
Adagrasib
RNK07421
Afatinib
Combination
F231
Control
Adagrasib
RNK07421
F671
Control
Adagrasib
RNK07421
Capmatinib
Combination
RLUN029

B

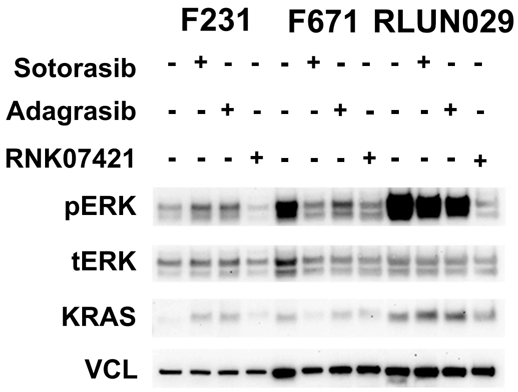
F231
F671
RLUN029
Sotorasib
Adagrasib
RNK07421
pERK
tERK
KRAS
VCL

Figure S7

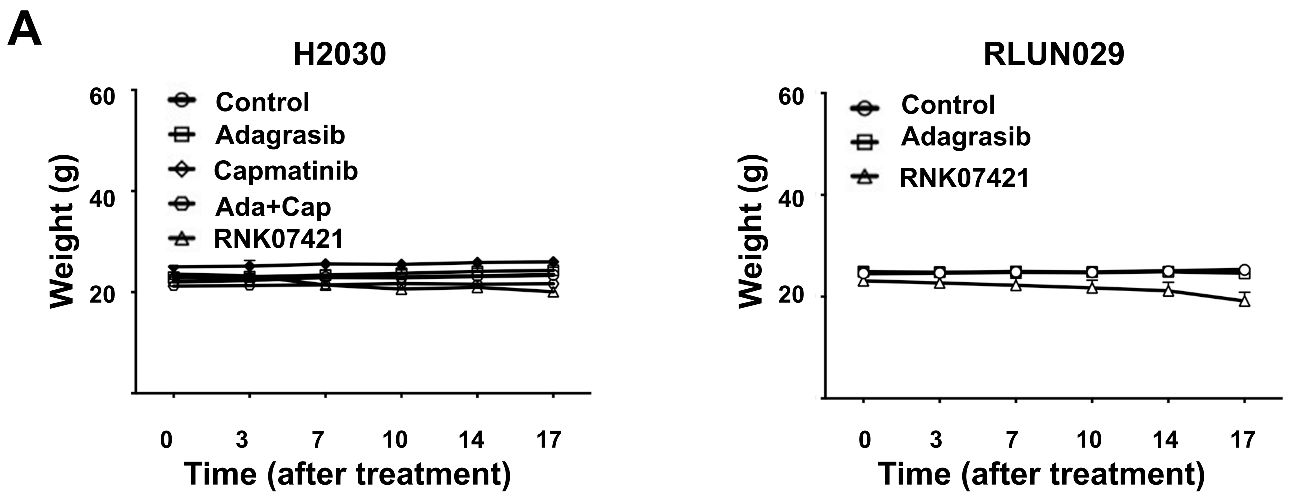
A
H2030
60
40
20
Weight (g)
Control
Adagrasib
Capmatinib
Ada+Cap
RNK07421
0
3
7
10
14
17
Time (after treatment)
RLUN029
60
40
20
Weight (g)
Control
Adagrasib
RNK07421
0
3
7
10
14
17
Time (after treatment)

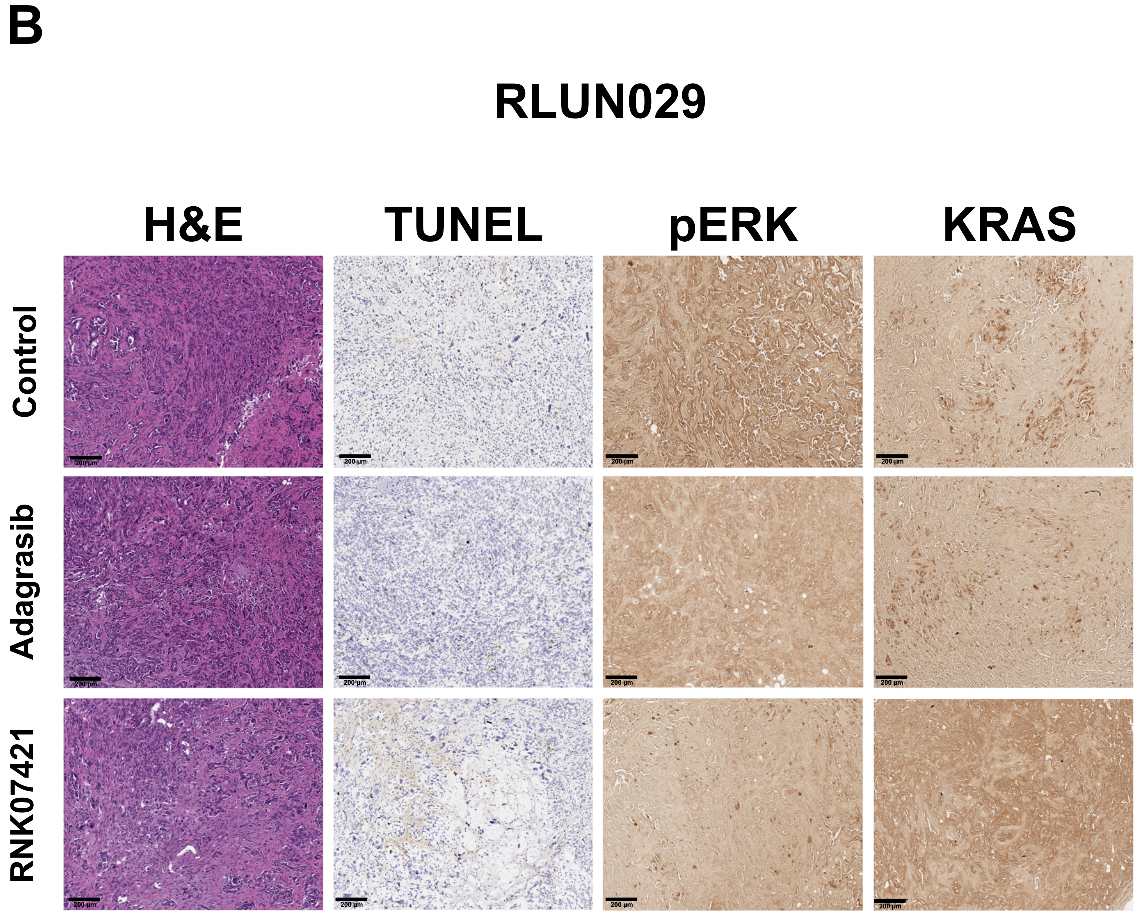
B
RLUN029
H&E
TUNEL
pERK
KRAS
Control
Adagrasib
RNK07421
