# Supplementary material for: Chaperone directed heterobifunctional molecules circumvent KRASG12C inhibitor resistance
Source: Cancer Lett. Author manuscript; Available in PMC 2026 Jun 18. (PMC13276862; doi:10.1016/j.canlet.2025.217691)
Supplement: 2 [file NIHMS2173876-supplement-2.docx]

**SUPPLEMENTARY MATERIAL AND METHODS:**

**NSCLC resistant cell lines**

H358 and MIA PaCa2 KRAS^G12C^ mutant cell lines were exposed to increasing concentrations of adagrasib or sotorasib over 6 months in a manner similar to previously reported [1, 2]. All resistant cells were able to proliferate normally in the presence of 10 μM G12C inhibitors. Occasionally, resistant cells were challenged with inhibitors to maintain drug resistance. Drug sensitivity was examined every regularly.

**Patient-derived organoids culture**

F231 and F671 were obtained from the NCI PDMR repository and maintained as specified. Organoids were grown in BME2 (#3533, R&D) domes until confluency was reached. The domes were then disaggregated with Dispase II (#17105041, ThermoFisher) for 2h at 37°C, organoids were collected and centrifuged for 5 minutes at 200g prior seeding in agarose microarrays for viability assays [3]. The PDOs were grown in complete media 6A (50% L-WRN conditioned media, 1.25mM N-acetylcysteine (#A9165, Sigma-Aldrich), 10mM nicotinamide (#N0636, Sigma-Aldrich), 1X N21 MAX Media Supplement (#AR008, R&D), 1X N-2 MAX Media Supplement (#AR009, R&D) and 10 µM Y-27632. (#1254, Tocris). RLUN029 was obtained from the Fukushima Medical School (Summit Pharmaceuticals). PDO sample was already established by Fukushima Medical School from de-identified NSCLC patients under their IRB [4-6].

**STR Profiling and Analysis**

DNA was extracted using the DNeasy Blood and Tissue Kit (#69504, Qiagen). DNA samples were quantified using NanoDrop ND-2000. Samples were submitted to the NUSeq Core Facility in the Center for Genetic Medicine at Northwestern University for human cell line authentication through STR profiling. The results were compared to known STR profiles for NSCLC cell lines available on the ATCC website (https://www.atcc.org/)

|  | **H358** | | **H358S-R** | | **H358A-R** | | **MIA PaCa-2** | | **MIA PaCa-2S-R** | | **MIA PaCa-2A-R** | |
| --- | --- | --- | --- | --- | --- | --- | --- | --- | --- | --- | --- | --- |
| **TH01** | 6 | 6 | 6 | 6 | 6 | 6 | 9 | 10 | 6 | 6 | 6 | 6 |
| **D21S11** | 28 | 30 | 28 | 30 | 28 | 30 | 29 | 31.2 | 28 | 30 | 28 | 30 |
| **D5S818** | 10 | 12 | 10 | 12 | 10 | 12 | 12 | 13 | 10 | 12 | 10 | 12 |
| **D13S317** | 8 | 12 | 8 | 12 | 12 | 12 | 12 | 13 | 8 | 12 | 12 | 12 |
| **D7S820** | 10 | 11 | 10 | 11 | 10 | 11 | 12 | 13 | 10 | 11 | 10 | 11 |
| **D16S539** | 12 | 13 | 12 | 13 | 12 | 13 | 10 | 13 | 12 | 13 | 12 | 13 |
| **CSF1PO** | 11 | 12 | 11 | 12 | 11 | 12 | 10 | 10 | 11 | 12 | 11 | 12 |
| **AMEL** | X | Y | X | Y | X | Y | X | X | X | Y | X | Y |
| **vWA** | 17 | 17 | 17 | 17 | 17 | 17 | 15 | 15 | 17 | 17 | 17 | 17 |
| **TPOX** | 8 | 9 | 8 | 9 | 8 | 9 | 9 | 9 | 9 | 9 | 9 | 9 |

|  | **H2030** | | **H1792** | | **H23** | |
| --- | --- | --- | --- | --- | --- | --- |
| **TH01** | 9.3 | 9.3 | 7 | 7 | 6 | 6 |
| **D21S11** | 30 | 30 | 31 | 31 | 30 | 30 |
| **D5S818** | 11 | 11 | 12 | 12 | 12 | 13 |
| **D13S317** | 12 | 12 | 12 | 12 | 12 | 12 |
| **D7S820** | 10 | 10 | 8 | 11 | 9 | 10 |
| **D16S539** | 12 | 12 | 10 | 11 | 11 | 11 |
| **CSF1PO** | 11 | 11 | 10 | 10 | 10 | 10 |
| **AMEL** | X | X | X | X | X | X |
| **vWA** | 15 | 15 | 14 | 16 | 16 | 17 |
| **TPOX** | 9 | 12 | 11 | 11 | 8 | 9 |

**List of antibodies**

| **Target** | **Application** | **Vendor** | **Catalog Number** |
| --- | --- | --- | --- |
| E-cadherin (24E10) | WB | CST | 3195 |
| H-RAS | WB | abcam | 18295-1-AP |
| HSF1 | WB | CST | 4356 |
| Hsp70 | WB | CST | 4872 |
| IRE1a | WB | CST | 3294 |
| KRAS (EPR23474-76) | WB/IHC | LSBio | LS‑C175665 |
| MEK 1/2 | WB | CST | 9126 |
| N-cadherin (D4R1H) XP | WB | CST | 13116 |
| N-Ras (F155) | WB | Santa Cruz Biotechnology | sc-31 |
| p44/42 MAPK (Erk 1/2) | WB/IHC | CST | 9102 |
| Phospho-EGFR (Tyr992) | WB | CST | 2235 |
| Phospho-HSF1 (Ser221) | WB | Aviva | OASG03625 |
| Phospho-HSF1 (Ser326) | WB | Life Technologies | BSM-52166R |
| Phospho-MEK 1/2 (Ser217/221) | WB | CST | 9154 |
| Phospho-p44/42 MAPK (Erk 1/2) (Thr202/Tyr204) | WB | CST | 9101 |
| RAS (E4K9L) | WB | CST | 91054 |
| Vimentin (D21H3) XP | WB | CST | 5741 |
| Vinculin (E1E9V) XP | WB | CST | 13901 |
| Anti-Mouse IgG, HRP-linked | WB | CST | 7076 |
| Anti-Rabbit IgG, HRP-linked | WB | CST | 7074 |

Abbreviations: WB: Western blot, IHC: Immunohistochemistry, CST: Cell Signaling Technology.

**RAS activity assay**

Cell lines were treated with vehicle or drug for two hours. Cells were then harvested, and RAS activity was assessed by GST-RAF-RBD pulldown using an Active Ras Detection Kit (#8821, Cell Signaling Technology) as per the manufacturer’s instructions with RAS antibody.

**Patient-derived organoids Cell Viability Assays**

Organoids were cultured in agarose microarrays [3] and treated 24 hours after seeding. Viability was evaluated 72 hours post-treatment. A staining solution of live cell dye calcein AM (C1430, ThermoFisher Scientific), propidium iodide (P3566, ThermoFisher Scientific) and Hoechst 33342 (H3570, ThermoFisher Scientific) was added to the organoids with a final concentration of 3 μg/mL. After a 30-minute incubation at 37 °C, live/ dead images of the spheroids were captured using an Olympus IX-83 fluorescence microscope and Zeiss laser scanning confocal microscope (LSM) 710. The live/dead images were analyzed to determine viability using the Olympus CellSens software by comparing the area of the propidium iodide signal with the area of the Hoechst signal for each organoid and by comparing the number of spheroids with propidium iodide signal to the number of spheroids with Hoechst signal for each spheroid array.

**CellTiter-Glo® Luminescent Cell Viability Assay**

A total of 3000 cells per well were seeded in 96-well plates. Different concentrations of RNK07421, sotorasib and adagrasib were tested in triplicates and after 72 hours CellTiter-Glo reagent was added according to manufacturer.

The software of GraphPad Prism 7.0 was used to calculate IC50.

**TaqMan Real-Time qPCR**

Total RNA was converted to cDNA using a High-Capacity RNA- to-cDNA Kit (#4387406, Thermo Fisher Scientific). Assays were conducted using 50 ng of cDNA, TaqMan Gene Expression Master Mixes and Gene Expression Assays (Thermo Fisher Scientific) with specific probes listed below. Human b-D-glucuronidase (GUSB) was used as an endogenous control. Samples were run in triplicate using a QuantStudio 6 Flex Real-Time PCR System and analyzed via the comparative ΔΔCt method. The following probes were used: FAM/ FAM/MGB–labeled against KRAS, HRAS and NRAS (Hs00364282_m1, Hs00978050_g1, Hs00180035_m1) respectively (Thermo Fisher Scientific).

**siRNA Transfection**

SiRNA (pooled oligos) was transfected into the target cells using TransIT-siQUEST reagent (#MIR 2114, Mirus) according to manufacturer’s instructions. The catalog numbers for the siRNAs used are as follows:

| **siRNA** | **Catalog number** |
| --- | --- |
| Control siRNA-A | Santa Cruz Biotechnology, sc-37007 |
| KRAS siRNA | Santa Cruz Biotechnology, sc-35731 |
| HRAS siRNA | Santa Cruz Biotechnology, sc-29340 |
| NRAS siRNA | Santa Cruz Biotechnology, sc-36004 |

**KRAS sequencing**

KRAS G12C and the acquisition of new KRAS mutations was evaluated. Total RNA was extracted, and cDNA was generated as described above. The KRAS coding sequence was amplified by PCR with F 5’-ATTTCGGACTGGGAGCGAG-3' and R 5'-GTCCTGAGCCTGTTTTGTGTC-3' primers. Then, the KRAS nucleotide sequence was analyzed by Sanger sequencing.

**Expression profiling of adagrasib resistance with RNA sequencing**

Gene expression profiling of acquired resistance to adagrasib was performed through NovaSeq6000 platform (Illumina), 25 million reads/sample. High-quality reads were aligned to the reference genome (GRCh38) using STAR. Differential gene expression analysis was performed with DESeq2 to identify genes that are significantly up- or down-regulated between groups.

**Gene Set enrichment analysis**

GSEA (http://www.broadinstitute.org/gsea) was used to determine the gene set enrichment of experimental signatures related with KRAS and mTORC1 Signaling, EMT, Protein secretion and E2F and MYC targets obtained from the MsigDB (<http://www.broadinstitute.org/gsea/msigdb>) [7]. GSEA estimates whether the members of a given gene signature are found at the top or bottom of a list of genes ranked by signal-to-noise ratio, indicating they are associated with a specific phenotype (e.g., KRAS-G12Ci resistance), rather than being distributed uniformly or randomly across the gene list. An enrichment score (ES) was calculated to quantify the degree to which a gene signature is overrepresented at the top or bottom of the entire ranked list. GSEA normalized the ES for each gene set to account for the variation in set sizes, yielding a normalized enrichment score (NES) and a false discovery rate (FDR). The FDR gave an estimate of the probability that a gene set with a given NES represents a false positive finding; it was computed by comparing the tails of the observed and empirical gene set-based permutated null distributions for the NES. The original expression data has been submitted to Gene Expression Omnibus (GEO submission in progress, accession number pending).

**Cellular perturbation analysis**

iLINCS (http://ilincs.org) directly matches transcriptional signatures of a given experimental condition (e.g., adagrasib resistance) with negatively correlated transcriptional signatures of chemical perturbations (CP) using the Connectivity Map (CMAP) approach to identify potential drug candidates that can reverse the phenotype [8]. We used iLINCS platform to calculate the connectivity of Adagrasib resistance signature (composed of up- and downregulated genes) by assigning −1 to downregulated and +1 to upregulated genes and calculating Pearson’s correlation between such vector and the library of transcriptional signatures of chemical perturbations. iLINCS include precalculated transcriptomic signatures of more than 15,000 chemicals.

**Heterobifunctional inhibitors**

Heterobifunctional compounds were generated by joining the KRAS^G12C^i Adagrasib, with the HSP90i, Luminespib, via chemical linkers optimized for attachment point on each inhibitor, length, and flexibility. Compounds were tested for cytotoxicity against a panel of KRAS^G12C^ cancer cell lines. Hetero functional groups were added to promising lead compounds to increase biological efficacy, retested, and selected.

| ID# | Parental G12Ci | Parental HSP90i |
| --- | --- | --- |
| RNK07311 | Adagrasib | Luminespib |
| RNK07421 | Adagrasib | Luminespib |

**RNK07311**


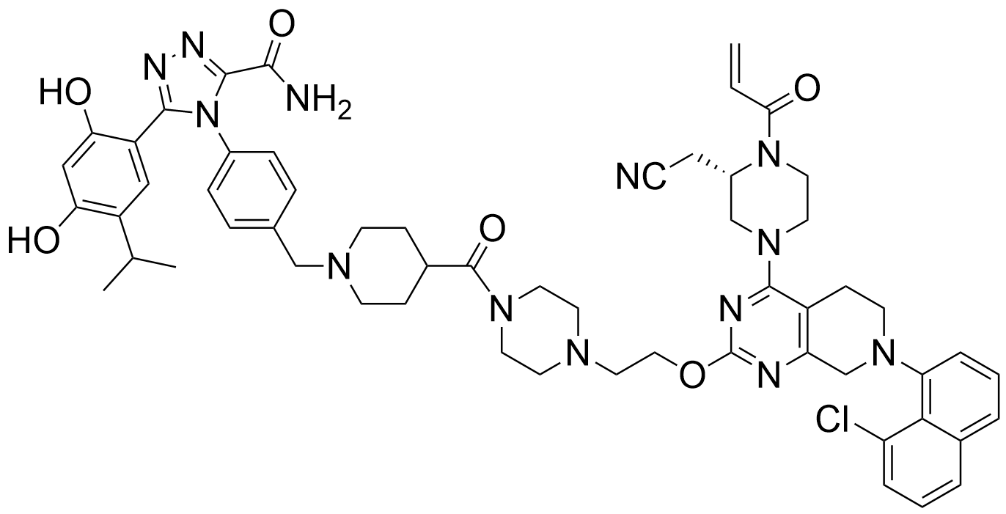


(S)-4-(4-((4-(4-(2-((4-(4-acryloyl-3-(cyanomethyl)piperazin-1-yl)-7-(8-chloronaphthalen-1-yl)-5,6,7,8-tetrahydropyrido[3,4-d]pyrimidin-2-yl)oxy)ethyl)piperazine-1-carbonyl)piperidin-1-yl)methyl)phenyl)-5-(2,4-dihydroxy-5-isopropylphenyl)-4H-1,2,4-triazole-3-carboxamide. 1H NMR (400 MHz, MeOD) δ 7.84 (d, J = 7.4 Hz, 1H), 7.72 (d, J = 8.1 Hz, 1H), 7.62 (d, J = 8.3 Hz, 2H), 7.57 – 7.47 (m, 4H), 7.41 – 7.32 (m, 2H), 6.85 – 6.70 (m, 2H), 6.36 –6.23 (m, 2H), 5.85 (d, J = 10.3 Hz, 1H), 4.45 – 4.26 (m, 5H), 4.06 – 3.74 (m, 6H), 3.71 – 3.54 (m, 6H), 3.52 – 3.39 (m, 6H), 3.26 – 3.19 (m, 2H), 3.15 – 2.92 (m, 7H), 2.87 – 2.77(m, 2H), 2.07 – 1.88 (m, 5H), 0.93 (d, J = 6.9 Hz, 6H). LC-MS: m/z 1062.4 [M+H]+.

**RNK07421**


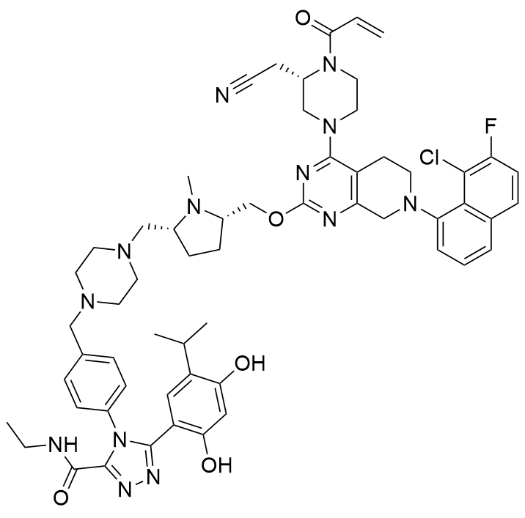


Detail on the synthesis of RNK07421:

To a stirred mixture of 2-[(2S)-4-[7-(8-chloro-7 -fluoronaphthalen-1-yl) -2-{[(2S,5R)-1 –methyl -5-(piperazin-1-ylmethyl)pyrrolidin-2-yl]methoxy}-5H,6H,8H-pyrido[3,4-d]pyrimidin-4-yl]-1-(prop-2-enoyl)piperazin-2-yl]acetonitrile (103 mg, 0.147 mmol, 1 equiv) and 5-(2,4-dihydroxy -5- isopropylphenyl)-N-ethyl-4-(4-formylphenyl)-1,2,4-triazole-3-carboxamide (86.77 mg, 0.220 mmol, 1.5 equiv) in DMF (4 mL, 51.686 mmol, 352.41 equiv) were added STAB (248.67 mg, 1.176 mmol, 8 equiv) in portions at room temperature under nitrogen atmosphere. The resulting mixture was stirred for 16 h at room temperature under nitrogen atmosphere. The reaction was quenched with water/ice at room temperature. The aqueous layer was extracted with CH_2_Cl_2_ (3x10 mL). The resulting mixture was concentrated under vacuum. The crude product (30 mg was purified by Chiral-Prep-HPLC with the following conditions: Column, C18, 30x150 mm, 5µm; mobile phase, water (10 mmol/L NH_4_HCO_3_) and ACN (45% ACN up to 80% in 10 min); Detector, uv 254 nm. This resulted in 4-{4-[(4-{[(2R,5S)-5- ({[7-(8- chloro-7- fluoronaphthalen-1-yl)-4-[(3S)-3-(cyanomethyl)-4-(prop-2-enoyl)piperazin-1-yl]-5H,6H,8H-pyrido[3,4-d]pyrimidin-2-yl]oxy}methyl)-1-methylpyrrolidin-2-yl]methyl}piperazin-1-yl)methyl]phenyl}-5-(2,4-dihydroxy-5-isopropylphenyl)-N-ethyl-1,2,4-triazole-3-carboxamide (13.82 mg, 8.31%) as a white solid.

LCMS (ES, *m/z*):1080 [M+H] ^+^

^1^H NMR (400 MHz, DMSO-*d*_6_) δ 10.59 (s, 1H), 9.73 (s, 1H), 8.94 (t, *J* = 5.9 Hz, 1H), 8.01 (dd, *J* = 9.0, 5.8 Hz, 1H), 7.79 (dd, *J* = 8.1, 4.2 Hz, 1H), 7.60 (dd, *J* = 9.0, 1.3 Hz, 1H), 7.52 (t, *J* = 8.1 Hz, 1H), 7.45-7.38 (m, 1H), 7.36 (d, *J* = 8.0 Hz, 2H), 7.28 (d, *J* = 8.1 Hz, 2H), 6.84 (s, 1H), 6.57 (s, 1H), 6.34 (s, 1H), 6.18 (d, *J* = 16.6 Hz, 1H), 5.77 (d, *J* = 10.6 Hz, 1H), 4.95-4.75 (m,1H), 4.40-4.00 (m, 6H), 3.83 – 3.72 (m, 2H), 3.47 (s, 4H), 3.19 – 3.04 (m, 8H), 2.89 (p, *J* = 6.8 Hz, 2H), 2.67 (p, *J* = 1.9 Hz, 2H), 2.38-2.32 (m, 10H), 2.19 (d, *J* = 8.6 Hz, 2H), 1.91-1.84 (m, 2H), 1.50 (s, 2H), 1.03 (t, *J* = 7.2 Hz, 3H), 0.80 (d, *J* = 6.9 Hz, 7H).

**Histology and immunohistochemistry**

All immunohistochemistry procedures were performed in the Research Histology Core University of Illinois Chicago (Chicago, USA). Tumors were embedded in paraffin, sectioned (5 μm thickness), and sections were placed on glass slides. The slides were deparaffinized in xylene, rehydrated in series of graded alcohols, and boiled with sodium citrate buffer (pH 6). Sections were incubated with primary antibodies specific for pERK and KRAS. For antibody detection, Envision detection system peroxidase/DAB (Dako) was used. Image acquisition was performed with an Aperio AT2 microscope.

**REFERENCES**

1. Soucheray, M., et al., *Intratumoral Heterogeneity in EGFR-Mutant NSCLC Results in Divergent Resistance Mechanisms in Response to EGFR Tyrosine Kinase Inhibition.* Cancer Res, 2015. **75**(20): p. 4372-83.

2. Becker, J.H., et al., *CXCR7 Reactivates ERK Signaling to Promote Resistance to EGFR Kinase Inhibitors in NSCLC.* Cancer Res, 2019. **79**(17): p. 4439-4452.

3. Luan, Q., et al., *Non-small cell lung carcinoma spheroid models in agarose microwells for drug response studies.* Lab Chip, 2022. **22**(12): p. 2364-2375.

4. Takahashi, N., et al., *Construction of in vitro patient-derived tumor models to evaluate anticancer agents and cancer immunotherapy.* Oncol Lett, 2021. **21**(5): p. 406.

5. Takahashi, N., et al., *An In Vitro System for Evaluating Molecular Targeted Drugs Using Lung Patient-Derived Tumor Organoids.* Cells, 2019. **8**(5).

6. Hayashita, Y., et al., *A polycistronic microRNA cluster, miR-17-92, is overexpressed in human lung cancers and enhances cell proliferation.* Cancer Res, 2005. **65**(21): p. 9628-32.

7. Subramanian, A., et al., *Gene set enrichment analysis: a knowledge-based approach for interpreting genome-wide expression profiles.* Proc Natl Acad Sci U S A, 2005. **102**(43): p. 15545-50.

8. Pilarczyk, M., et al., *Connecting omics signatures and revealing biological mechanisms with iLINCS.* Nat Commun, 2022. **13**(1): p. 4678.

**List of abbreviatures**

DMSO: Dimethyl sulfoxide

DNA: Deoxyribonucleic acid

EGFR: Epidermal growth factor receptor

FC: Fold change

FDA: Food and drug administration

FDR: False Discovery Rate

GIST: Gastrointestinal stromal tumors

GSEA: Gene set enrichment analysis

HSP90: Heat-shock protein 90

HTRF: Homogeneous time-resolved fluorescence

IC50: Half maximal inhibitory concentration

KRAS: Kirsten rat sarcoma viral Homologue

mRNA: Messenger ribonucleic acid

NSCLC: Non-small cell lung carcinoma

NSG: NOD scid gamma

PDMR: Patient-Derived Models Repository

PDMS: Polydimethylsiloxane

PDO: Patient-derived organoid

RNA: Ribonucleic acid

RNA-seq: RNA sequencing

RT-qPCR: Quantitative reverse transcription polymerase chain reaction

S.D.: Standard deviation

RTK: Receptor tyrosine kinase

VEGFR-2: Vascular endothelial growth factor receptor 2
